# *In silico* Description of LAT1 Transport Mechanism at an Atomistic Level

**DOI:** 10.3389/fchem.2018.00350

**Published:** 2018-08-24

**Authors:** Luca Palazzolo, Chiara Parravicini, Tommaso Laurenzi, Uliano Guerrini, Cesare Indiveri, Elisabetta Gianazza, Ivano Eberini

**Affiliations:** ^1^Dipartimento di Scienze Farmacologiche e Biomolecolari, Università degli Studi di Milano, Milan, Italy; ^2^Dipartimento di Biologia, Ecologia e Scienze della Terra, University of Calabria, Cosenza, Italy

**Keywords:** LAT1, targeted molecular dynamic, aminoacid transporters, tyrosine, molecular docking

## Abstract

The molecular mechanism of transport mediated by LAT1, a sodium-independent antiporter of large neutral amino acids, was investigated through *in silico* procedures, specifically making reference to two transported substrates, tyrosine (Tyr) and leucine methyl ester (LME), and to 3,5-diiodo-L-tyrosine (DIT), a well-known LAT1 inhibitor. Two models of the transporter were built by comparative modeling, with LAT1 either in an outward-facing (OF) or in an inward-facing (IF) conformation, based, respectively, on the crystal structure of AdiC and of GadC. As frequently classic Molecular Dynamics (MD) fails to monitor large-scale conformational transitions within a reasonable simulated time, the OF structure was equilibrated for 150 ns then processed through targeted MD (tMD). During this procedure, an elastic force pulled the OF structure to the IF structure and induced, at the same time, substrates/inhibitor to move through the transport channel. This elastic force was modulated by a spring constant (*k*) value; by decreasing its value from 100 to 70, it was possible to comparatively account for the propensity for transport of the three tested molecules. In line with our expectations, during the tMD simulations, Tyr and LME behaved as substrates, moving down the transport channel, or most of it, for all *k* values. On the contrary, DIT behaved as an inhibitor, being (almost) transported across the channel only at the highest *k* value (100). During their transit through the channel, Tyr and LME interacted with specific amino acids (first with Phe252 then with Thr345, Arg348, Tyr259, and Phe262); this suggests that a primary as well as a putative secondary gate may contribute to the transport of substrates. Quite on the opposite, DIT appeared to establish only transient interactions with side chains lining the external part of the transport channel. Our tMD simulations could thus efficiently discriminate between two transported substrates and one inhibitor, and therefore can be proposed as a benchmark for developing novel LAT1 inhibitors of pharmacological interest.

## Introduction

The L-type amino acid transporter 1 (LAT1, SLC7A5) is a ubiquitous Na^+^- and H^+^-independent antiporter, involved in cellular uptake of essential amino acids. Being over-expressed in many human cancers that are characterized by an increased demand of amino acids, it was recently acknowledged as a novel target for cancer therapy (Haase et al., 2007; Napolitano et al., 2017b). LAT1 is also expressed in the blood-brain barrier (BBB), where it allows the transit of essential amino acids and also of some drugs, including the antiparkinson drug L-dopa and the anticonvulsant gabapentin (Dickens et al., 2013; Scalise et al., 2018). Natural mutations of LAT1 impair brain cells amino acid uptake and have been linked to Autism Spectrum Disorder (ASD) (Tărlungeanu et al., 2016). For these reasons, much attention is currently devoted to LAT1 transporter activity (Napolitano et al., 2017a,b; Scalise et al., 2018), and to its inhibition, e.g., with 3,5-diiodo-L-tyrosine (DIT), fenclonine, and acivicine (Geier et al., 2013; Schlessinger et al., 2013).

**Figure 1:**
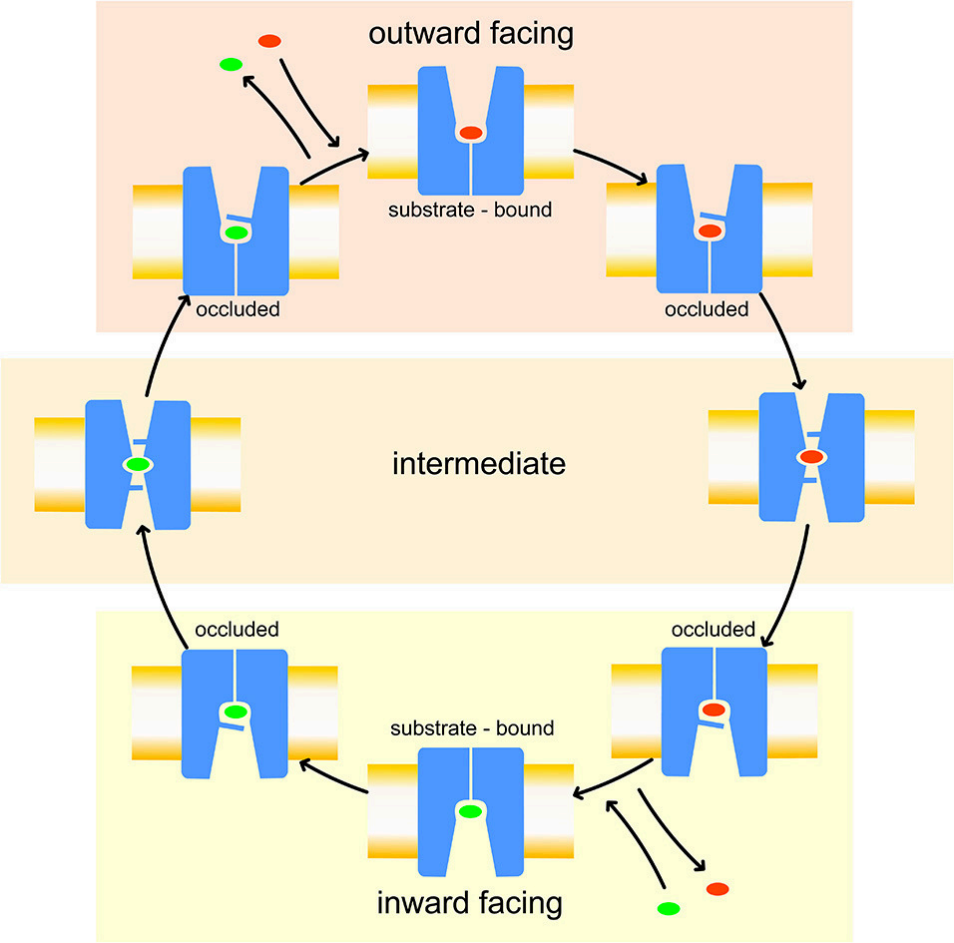
Leu T fold proposed model of transport. Red and green ovals represent the substrates transported, respectively, from extracellular to intracellular side and *vice-versa*.

Like other members of the amino-acid-polyamine-organocation (APC) family (Napolitano et al., 2017a), LAT1 is characterized by 12 transmembrane α-helices, which can be grouped in three different domains (Boudker and Verdon, 2010; Diallinas, 2014): α-helices 1–5 and 6–10 are involved in the transport mechanism and have an antiparallel symmetry in their secondary structures, while 11 and 12 are necessary for the homo-dimerization of the transporter. The repetitive symmetry of the first two domains gives to the α-helices a discrete mobility within the cell membrane and defines the binding site for transported substrates, in the middle of the transport channel. This structural feature is defined “LeuT fold,” from the name of the first crystallized transporter belonging to the APC family (Krammer et al., 2016; Kazmier et al., 2017). For LeuT fold proteins, a model of transport has been proposed: in the initial phase of the process, the protein lies in a “outward-facing” (OF) conformation and receives the transported substrate in its binding site. The interaction between protein and transported substrate promotes the gate closure: this starts from the rotation of a specific conserved aromatic amino acid (see below), which in turn leads to a conformational change in the whole protein, promoting its transition to an “inward-facing” (IF) conformation (Gao et al., 2010; Krammer et al., 2016). Afterward, the substrate is released by the transporter in the intracellular space and the protein, with a new substrate to be brought to the extracellular space, returns to its OF conformation, passing through intermediate transitory occluded states, as shown in Figure 1.

The details of this mechanism are not yet clarified, as the few APC crystallographic structures so far resolved are either in OF (AdiC, LeuT, and BetP) or in IF (Mhp1 and GadC) conformations, and none is in a non-occluded/occluded intermediate state (Singh et al., 2008; Weyand et al., 2008; Quick et al., 2009; Ressl et al., 2009; Zhou et al., 2009; Gao et al., 2010; Kroncke et al., 2010; Shimamura et al., 2010; Kowalczyk et al., 2011; Perez et al., 2011; Ma et al., 2012; Koshy et al., 2013; Simmons et al., 2014; Ilgü et al., 2016). In this context, molecular modeling, based on comparative modeling together with non-standard molecular dynamics (MD) simulations, such as targeted molecular dynamics (tMD), steered molecular dynamics (sMD) or meta-dynamics (MTD), has been widely exploited to shed light on the APC transport mechanism. For instance, in AdiC, an arginine/agmatine antiporter of *E. coli*, the interactions between the transported substrate and the side chains that define the transport channel have been studied in depth, through non-standard molecular dynamics simulations (Krammer et al., 2016), to identify the pivotal residues in the process (Shaikh and Tajkhorshid, 2010; Cheng and Bahar, 2013, 2014; Geier et al., 2013; Zhao and Noskov, 2013; Stolzenberg et al., 2015; Krammer et al., 2016; Chiu et al., 2017).

For LAT1, a specific phenylalanine (Phe252) has been proposed as the “upper gate,” since its closure is involved it the transition from OF to IF.

Despite its biochemical and pharmacological interest, LAT1 structure has not been solved yet by crystallography. Some LAT1 comparative models have already been proposed, mostly based on the crystallographic structure of AdiC in the OF conformation (Dickens et al., 2013; Geier et al., 2013; Augustyn et al., 2016; Colas et al., 2016; Napolitano et al., 2017a; Ilgü et al., 2018) which covers both the open and closed LAT1 conformations. However, since these models are all based on the only available OF conformation of different AdiC crystallographic structures, it is currently unknown which residues are actually involved in the substrate transport when LAT1 is in its IF conformation.

To overcome this limitation, in the present work we performed 12 different tMD simulations, involving two transported substrates, tyrosine (Tyr) and leucine methyl ester (LME), and a transport inhibitor, 3,5-diiodo-L-tyrosine (DIT) (Geier et al., 2013; Zur et al., 2016), and discussed our findings. With this investigation, we meant: (i) identifying the key residues for the transport along the whole channel, (ii) comparing the transport mechanism of molecules characterized by opposite functional effects on LAT1 and (iii) providing the first *in silico* evidence of a LAT1 inner gate to guide further *in vitro* experiments.

## Materials and methods

### Comparative modeling of LAT1

LAT1 comparative modeling was based on already published multiple alignments (Napolitano et al., 2017a,b) of some members of the APC superfamily, including three amino acid/poly-amine antiporters [AdiC, CadB and PotE], one cationic amino acid transporter (CAT) [CAT6], one amino acid/choline transporter (ACT) [Uga4], one glutamate/GABA antiporter (GGA) [GadC], LAT1 and LAT2 (see Supplementary Material). Multiple alignment was carried out by the program Clustal Omega (Li et al., 2015).

According to their crystallization state, the crystallographic structures of AdiC (PDB ID: 3OB6), with Trp202 gate residue in an open conformation, and GadC (PDB ID: 4DJI) were selected as templates for modeling LAT1 OF and IF conformations, respectively. Selected 3D structures were first optimized and refined for further computational steps by correcting crystallographic-related errors and/or by filling up any unresolved residues (e.g., some amino acids in the GadC C-terminus) through the MOE Structure Preparation Module of the Molecular Operating Environment 2016.08 (MOE, Chemical Computing Group, Montreal, Canada). Comparative models were produced with the MOE Homology Model program with default settings, using the Amber10:EHT force field. Ten intermediate models were built and refined for each LAT1 conformation. Final models were selected according to the electrostatic solvation energy calculated with the Generalized Born/Volume Integral (GB/VI) methodology. The Ramachandran plot, the side chain packing, and the stereo chemical quality of the selected structures were checked with the MOE Protein Geometry module in order to verify that all these parameters were consistent with typical values found in crystal structures.

### Molecular dynamics simulation

The Desmond Molecular Dynamics System (D. E. Shaw Research, New York, NY, 2018, Schrödinger, New York, NY, 2018) system builder tool was used to place the OF LAT1 model into a POPC membrane bilayer. LAT1 orientation was set up according to the OPM server (Lomize et al., 2012), which provides spatial arrangements of membrane proteins with respect to the hydrocarbon core of the lipid bilayer. The N- and C-termini of the protein were capped. The system was solvated with 10,174 TIP3P water molecules in a cubic box with 90 Å edges; the exceeding positive charge was neutralized by adding chloride ions; sodium chloride was added up to 0.1 M concentration. The system was energy-minimized down to a root mean square (RMS) gradient of 0.05 kcal/mol/Å^2^ using the MOE software, to relax the system and remove clashes between protein, membrane and solvent in the new setup.

To produce an equilibrated model of OF LAT1, the system was submitted to a 150 ns molecular dynamics simulation (MD) using NAMD, prepared through the MOE graphical interface (Phillips et al., 2005), under periodic boundary conditions (PBC). The following parameters were set: 300 K and Langevin thermostat for temperature coupling, 1 bar and Nosé-Hoover Langevin piston for pressure coupling, 2 fs as integration time step. Coordinates and velocities of each atom were saved every 1 ps. The Amber10:ETH force field with the reaction-field treatment for electrostatics was applied in all the computational procedures. Analyses of the trajectories were performed with the VMD tool (Humphrey et al., 1996).

### Targeted molecular dynamics simulations

Targeted molecular dynamics (tMD) is a method to observe large-scale transitions between two known end-point conformations of a molecule, initial and final (target). To this purpose, a restraint energy term is added to the total energy of the system: frame-by-frame, each specified atom is subject to a harmonic potential (i.e., a spring) that pulls it toward its target position. The restraint energy term is assumed to be proportional to the square the difference between (a) the RMSD from instant to final coordinates and (b) the RMSD that there would be from instant to final coordinates assuming a linear transition between the initial and target coordinates. As with a harmonic oscillator, a constant of proportionality *k* (i.e., spring constant) expresses how strong is the force with which the system is pulled toward its target coordinates (Schlitter et al., 1994).

The LAT1 model coming from the equilibrating MD phase was energy minimized and used as the starting state, whereas the final state was generated by replacing the OF LAT1 model embedded into the membrane with the IF model.

Transported substrates and inhibitor were added to the systems at the top of the simulation box in the starting states, aligning them along the Z-axis of the transport channel. The solvent bulk was then energy-minimized down to a RMS gradient of 0.05 kcal/mol/Å^2^, using the MOE software and keeping protein, substrates/inhibitor and membrane atoms fixed.

To preserve the optimized atomic configuration coming from the model based on AdiC structure, only the α-carbon positions of LAT1 and the atoms of substrates/inhibitor were set as targeted atoms to guide the OF/IF transition, while side chains were set as freely moving.

**Figure 2:**
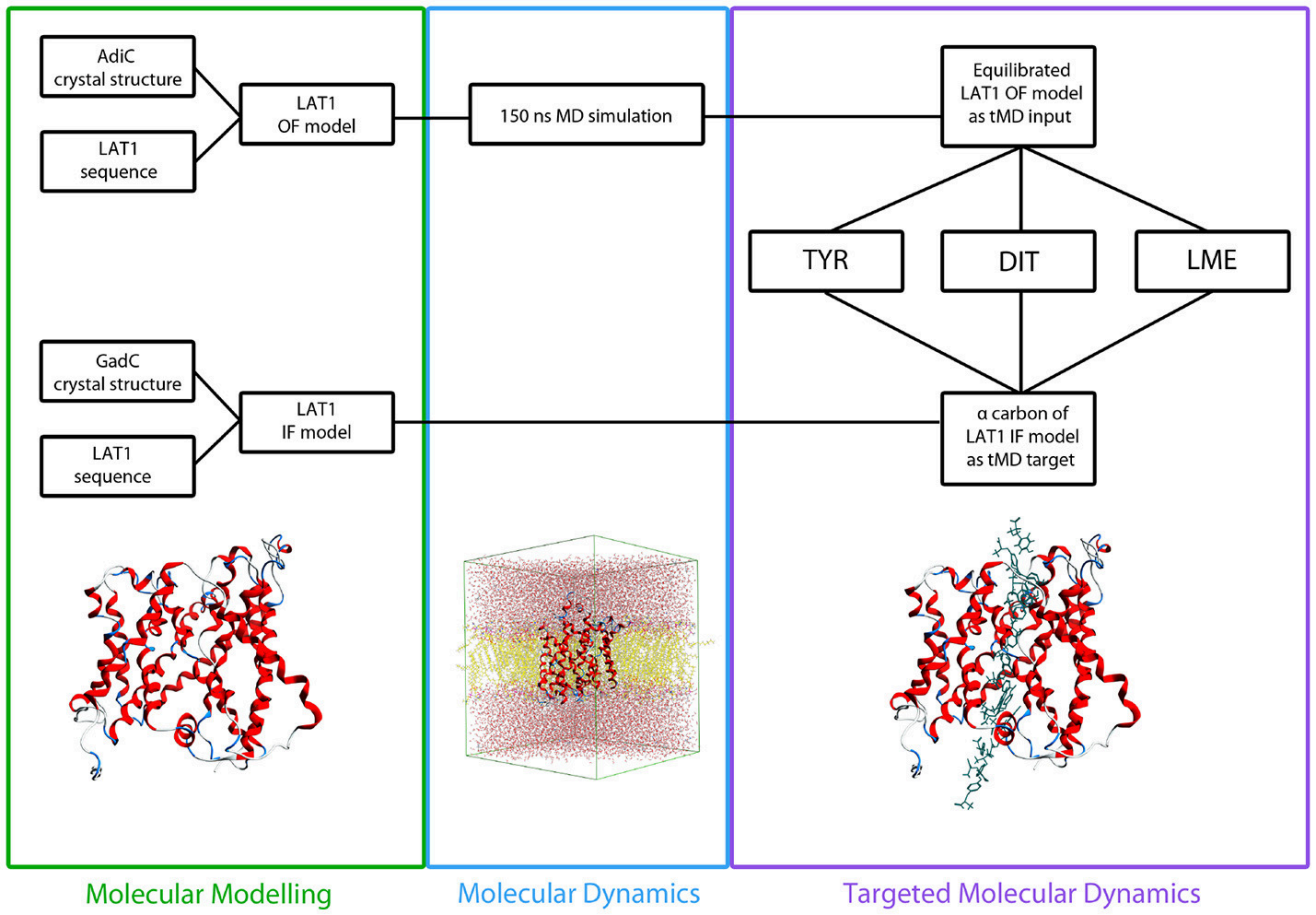
Flowchart of *in silico* set up. LAT1 OF and IF models were built starting from AdiC and GadC crystal structures, respectively, *via* homology modeling. LAT1 OF model was equilibrated through a 150 ns MD simulation and then used as starting structure for all the tMD simulations. α-Carbons of LAT1 IF model were used as targets for all the tMD simulations.

A total of 12 different tMDs were performed, four with Tyr, four with LME and four with DIT. Performed tMD simulations and their identifiers are listed in Table 1. In detail, for each investigated compound the same tMD simulations were carried out under the following conditions: (i) spring constant *k* = 100 for 10 ns, (ii) sequential 5 ns-tMD with decreasing values of *k* (*k* = 90, 80, and 70). The spring constant *k* controls the magnitude of the elastic potential that pulls each atom toward its final coordinates (Phillips et al., 2005). A schematic flowchart of the in silico set up is shown in Figure 2.

**Table 1:** tMD simulation identifiers.

**Identifier**	**Substrates/inhibitor**	**Time length [ns]**	***k***
T1	Tyr	10	100
T2	Tyr	5	90
T3	Tyr	5	80
T4	Tyr	5	70
L1	LME	10	100
L2	LME	5	90
L3	LME	5	80
L4	LME	5	70
D1	DIT	10	100
D2	DIT	5	90
D3	DIT	5	80
D4	DIT	5	70

The following parameters were set: 300 K and Langevin thermostat for temperature coupling, 1 bar and Nosé-Hoover Langevin piston for pressure coupling, 2 fs as integration time step. Coordinates and velocities of each atom were saved every 0.5 ps. Amber10:ETH was set as force field for all tMD simulations. All the tMD simulations were carried out using NAMD and prepared through the MOE graphical interface (Phillips et al., 2005).

### tMD analyses

Aqua-Duct (v. 0.3.7 - http://www.aquaduct.pl) was used to analyze the trajectories of the substrates/inhibitor across the transporter. For our calculations, we tracked every molecule passing through the LAT1 channel, defined as a spherical zone of radius 6 Å, centered on residues Thr109, Val190, Asn273, Leu385, and Phe451. All the trajectories were also analyzed using GROMACS analysis tools, supported by the VMD plugins. Trajectory time frames were clustered on the basis of Tyr, LME, and DIT positions relative to the LAT1 transport channel according to the simple linkage methodology. Reported data refer to the centroids of representative clusters. In order to compute the contribution of individual residues to the total interaction energy, a MOE svl script based on the interaction force fingerprint (IFFP) was applied. This method partitions the native force-field potentials and derives the residue-specific interaction forces of the ligand-receptor complex characteristic of hydrogen bonding, steric clashes, and other aspects of molecular interactions. The sum of these forces *per* residue provides the overall interaction of each single amino acid with the ligand and allows to compute the total interaction energy associated to the complex. It specifically works by subtracting non-bonded interactions of the ligand with each amino acid residue and using the resulting force vectors to describe the slope of the remaining potential (Shadnia et al., 2009).

### Molecular docking simulations

Resulting tMD trajectories were clustered, and the representative conformations (i.e., centroids of representative clusters) were used as LAT1 structures for molecular docking simulations (Jurik et al., 2015; Colas et al., 2016).

The binding sites of representative conformations were identified through the MOE Site Finder program. Molecular docking simulations of both substrates and inhibitor were carried out with the MOE Docking program with default settings, as previously described (Galli et al., 2014). Briefly, a multi stage docking framework was applied, according to the MOE Docking algorithm, which includes conformational search, placement, initial scoring, refinement and final scoring. The protein was treated as a rigid body, while intermolecular bonds of docked compounds were allowed freely rotation to generate preferred conformations prior to the placement stage. The Amber10:EHT force field was applied for both protein and substrates/inhibitor parametrization.

Depending upon the docking stage, docked compounds were ranked according to two different empirical scoring functions which provide an estimation of the free energy of binding (ΔG) and include in their calculation, among others contributions, solvation and entropy terms, or enthalpy terms based on polar interaction energies. In detail, the London dG scoring function and the forcefield-based GBVI/WSA ΔG scoring function were used for the initial and the final scoring of the docking poses, respectively (Naïm et al., 2007; Labute, 2008). More negative values of this parameter, expressed in kcal/mol, indicate a gain, less negative values a worsening in affinity for the substrate (Sensi et al., 2014; Platonova et al., 2017).

### *In silico* site-directed mutagenesis

Single point mutations were generated with the MOE Residue Scanning tool; the selected residues were mutated to alanine. To obtain more accurate results, the sites being mutated were treated as flexible by applying a conformational sampling method to both the target amino acid and the surrounding residues and generating an ensemble of conformations. Then, for each analyzed property of the protein ensemble monitored through the conformational search (i.e., protein thermostability and binding affinity for the substrate), average values were computed using a Boltzmann distribution. In detail, the Low Mode MD sampling method was selected for each *in silico* mutation to generate 25 conformational ensembles and to sample interactions between substrate and surrounding residues. To account for the effect of mutation on Tyr transport, relative thermostability (ΔStability) and binding affinity (ΔAffinity), with respect to the wild type LAT1, were computed as the Boltzmann average of the relative stabilities/affinities of the ensemble.

The change in protein stability due to mutation was computed through the MOE protein stability scoring function, trained on over 3000 single point mutations taken from the FoldX (Guerois et al., 2002) and PoPMuSiC-2.0 (Dehouck et al., 2009) datasets. This method is based on the prediction of the difference in stability (*s*) between the mutant and wild type in the folded (*f*) and unfolded (*u*) states of the protein, expressed as ΔΔ*G*_*s*_, computed as follow:

$$\begin{array}{l} \Delta \Delta G_{s}=\Delta G_{f}^{Mut}-\Delta G_{f}^{WT}=\Delta G_{sf}^{WT\to Mut}-\Delta G_{su}^{WT\to Mut} \end{array}$$

Accordingly, a model based on Linear Interaction Energy is applied, in which Δ*G* can be expressed as the change in the residue environment interaction energy going from wild-type to mutant, where the interaction energy is the change between the energy of the protein and the energy of the protein without the residue of interest.

The GBVI/WSA dG scoring function, an AMBER/GBVI-based scoring function trained on 99 protein-ligand complexes (Corbeil et al., 2012), was used to calculate the differences in affinity for the transported solute substrate Tyr upon mutations.

More negative values of ΔStability and ΔAffinity, expressed in kcal/mol, indicate increased stability of the transported structure and gain of affinity for the substrate. Conversely, more positive/less negative values of both parameters indicate loss in complex stability and worsening of affinity.

## Results and discussion

### OF and IF LAT1 models

LAT1 comparative models were built from multiple alignments of a set of transporters belonging to the APC family, as previously described (Napolitano et al., 2017a) and reported in Supplementary Material. Despite the existence of a binding partner of LAT1, SLC3, most of the published wet transport data have been obtained on the monomeric form of LAT1 (Napolitano et al., 2017a), which was demonstrated to exert its activity also in this form. For this reason, to characterize the transport mechanism of LAT1, we focused only on its monomeric form. In order to build LAT1 models in two different conformations, the crystallographic structures of known APC transporters were analyzed and compared by structural superposition.

Making reference to our sequence alignment (Napolitano et al., 2017a), as reference structure to model LAT1 in its OF conformation we selected AdiC, which, even with a sequence identity of 21% and similarity of 34%, represents the most suitable template available for modeling LAT1.

Membrane proteins are hard targets for structural biology due to the intrinsic instability of their crystals. Despite the number of solved structures has exponentially grown in the last few years, comparative modeling is still the method of choice if the unknown protein shares any significant sequence similarity with an experimental structure. However, the identification of topological fingerprints and sequence similarity networks has proven useful for improving the accuracy of low- (<30% sequence identity) or mid-range (<50%) comparative models (Schlessinger et al., 2013). In this respect, as already done for GPCRs (Isberg et al., 2016), also for some membrane transporters a numbering scheme has been proposed, providing common reference points within the topology, useful for guiding homology modeling and structural comparison across the entire family (Lee et al., 2016).

Among the few homologous transporters solved so far in IF state, namely ApcT and GadC (Shaffer et al., 2009; Ma et al., 2012), the latter shows a more clearly developed IF conformation than ApcT. This conclusion is supported by the higher RMSD values obtained when AdiC and GadC are superposed by structural alignment (6.1 Å), compared to those obtained by superposing AdiC and ApcT (4.9 Å). Moreover, α-helix 1, which is involved in the conformational changes in the transition from the OF to the IF state, is fully inward open in GadC (Supplementary Figure 1). On this basis, despite the low sequence identity, GadC was selected as the most suitable template to model LAT1 in IF conformation. However, this model was not used in any tMD simulations, but just to define the reference final (target) position of the α-carbons.

Protein geometry has been checked for the two crystallographic structures used as templates, and for the OF and IF models. The checked parameters are not significantly different between experimental (Supplementary Figures 2A,B) and computational structures (Supplementary Figures 2C,D).

**Figure 3:**
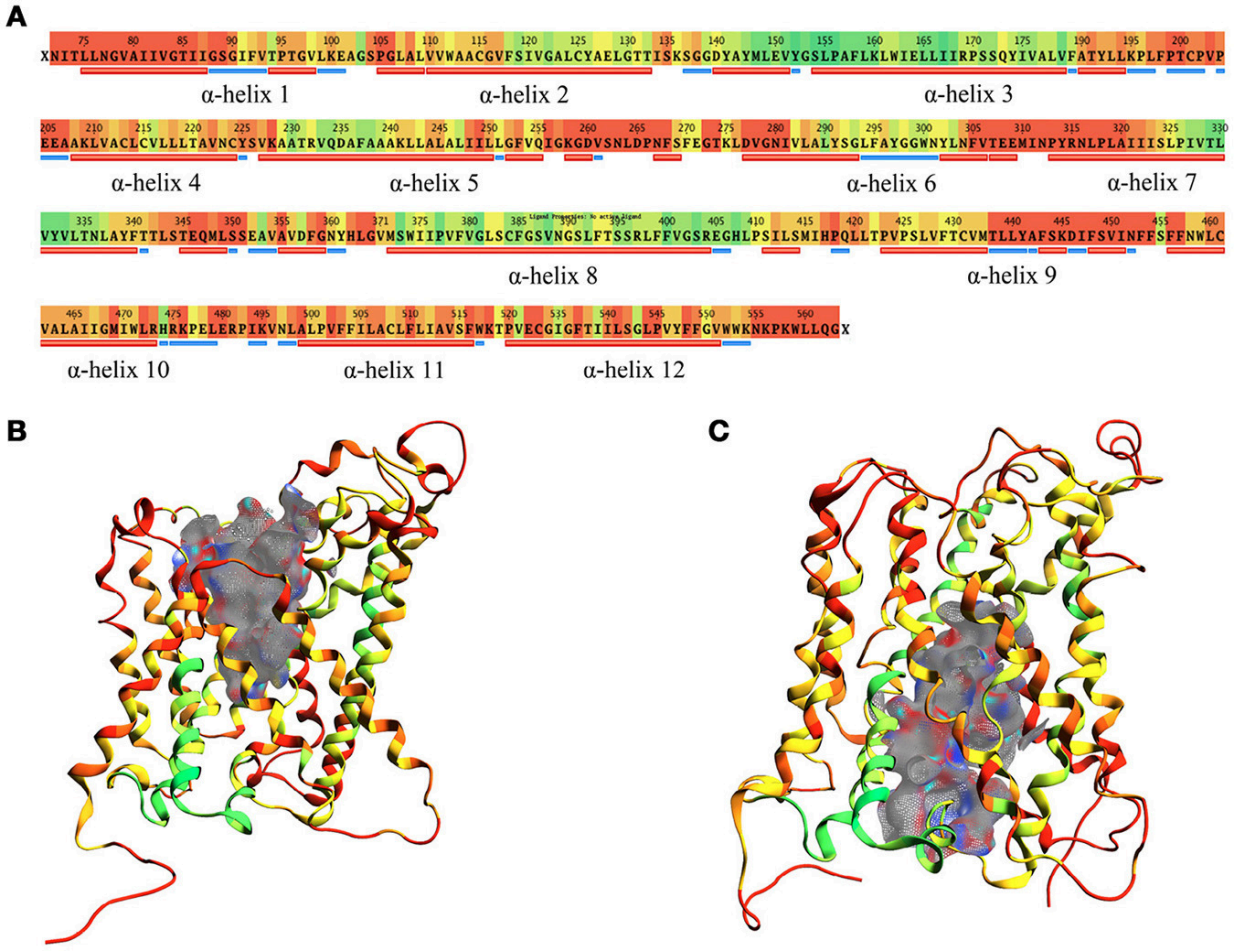
RMSD between OF and IF LAT1 models, computed for the α-carbons. **(A)** shows per-residue RMSD along LAT1 primary and secondary structure. Red corresponds to the maximal, green to the minimal RMSD values; LAT1 transport channel in OF and IF models are shown in **(B,C)**; ribbons are colored as per RMSD value.

The structural comparison between the two models allows pointing out the regions with high mobility and the ones less involved in conformational changes during the transport cycle. The RMSD between OF and IF conformation amounts to approximately 7.5 Å overall and is color coded on a residue-to-residue basis in Figure 3A. In agreement with previous literature data on homologous transporter structures (Krammer et al., 2016), the α-helices 1, 4, 11, and 12 of our models show a high RMSD value (average 6.5 Å) along their whole length; α-helices 2, 6, and 7 also show a high RMSD value (average 6.8 Å), but this is associated to conformational rearrangements in only a portion of their length, suggesting that the α-helices are involved to a different extent in this structural transition.

Two different transport channel surfaces were identified in OF (Figure 3B) and IF (Figure 3C) LAT1 models. In particular, in the OF model the transport channel surface is open to the extracellular side of the membrane; conversely, it is closed in its lower portion, mainly by residues Tyr259, Thr345, and Asn258 (Supplementary Figure 3A). On the other hand, the IF structure has a well-defined channel, open to the intracellular side of the membrane; the main residues that close the upper portion of the channel are Ile147, Phe252, Ser143, and Phe69 (Supplementary Figure 3B).

To ensure the robustness of our procedure, the stability of the OF model, used as reference structure for the following steps, was carefully assessed *via* 150 ns of MD simulation. To this end, the system was relaxed and the OF LAT1 structure equilibrated in an explicit membrane bilayer. Besides protein geometry quality validation, the general stability of the OF LAT1 model is also confirmed by the short-simulated time necessary for the protein to reach a RMSD plateau, characterized by a value (<3 Å) typical of stable proteins (Supplementary Figure 4). As expected, the largest root mean square fluctuation (RMSF) computed for the α-carbons is associated with the C-terminus (residues 437-441), a typical protein end-effect. The extracellular loops, residues 117-119 and 175-180, are associated to a RMSF of approx. 4 Å. Conversely, α-helices RMSF is <1 Å, suggesting very low flexibility for the transmembrane barrel structure. Our MD simulation thus suggests that the structure obtained from this equilibration phase is stable and it is also equivalent to the OF LAT1 structure already published in our previous works (Napolitano et al., 2017a,b) in which we validate our OF LAT1 model *via in vitro* experiments.

For these reasons, the equilibrated OF LAT1 model represents a suitable starting point for tMD simulations, supporting its use as a reference structure to guide LAT1 from an OF to an IF conformation.

### Differences in substrates and inhibitor transport mechanisms with *k* = 100

Frequently, classical MD simulations do not efficiently explore the conformational space and fail to reproduce large functional protein rearrangements. To bypass this limitation, tMD simulations were carried out. To model the transition from OF to IF LAT1 conformation within this protocol, the α-carbon positions of the IF LAT1 model were taken as reference, disregarding the side chains; this selection avoids any potential issue connected with the use of a distant homologous template. Accordingly, IF LAT1 model was never used in any of our tMD simulations.

The use of different experimental setups in tMD allowed us to discriminate the ability of the investigated compounds (Tyr, LME and DIT) either to be transported or to inhibit LAT1 activity, as well as to capture the complete transition from OF to IF resulting in the passage of substrates across the transport channel.

In detail, a simulation at a the relatively high spring constant value (*k* = 100 for 10 ns) was useful to assess the whole transport cycle and to allow the passage of all substrates/inhibitor through the LAT1 channel; decreasing *k* values (90, 80, 70, for 5 ns) were set to identify the conditions at which transport is allowed or blocked for substrates and inhibitor and to appreciate differences in their transport mechanism (Table 1).

Supplementary Movie 1 shows the entire transport simulation of the three compounds. Their paths during the tMD simulations were traced and are reported in Supplementary Figure 5. This analysis shows that, at the highest spring constant value, Tyr, LME and DIT are able to pass through the LAT1 transport channel, moving from the extracellular to the intracellular side of the membrane.

**Figure 4:**
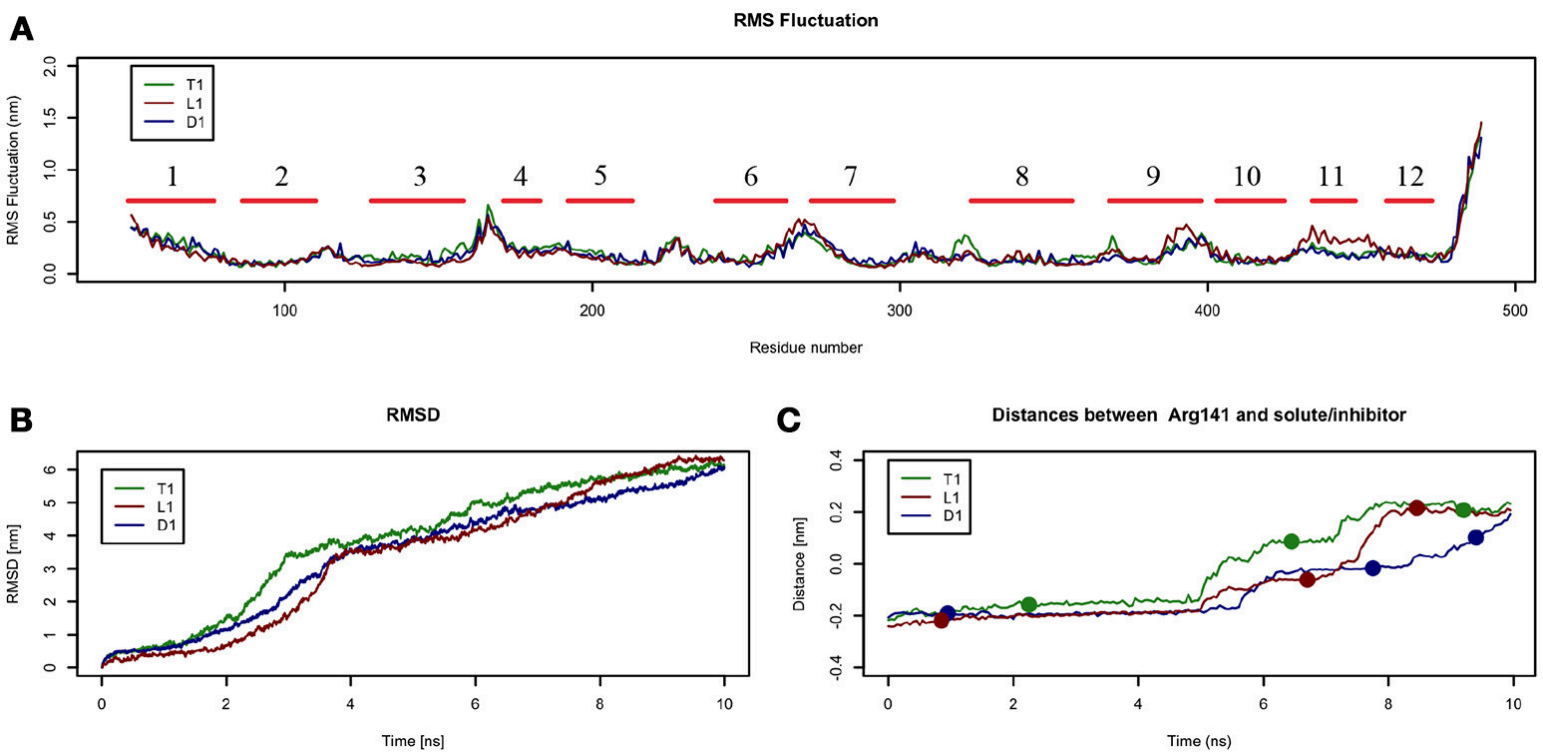
Geometrical changes of LAT1 during 10 ns tMD simulations. **(A)** shows the superposition of RMS fluctuation for both dynamics, computed for the α-carbons of LAT1; α-Helices are represented as red lines. **(B)** shows RMSD values over time for LAT1 during OF to IF transition in presence of Tyr, LME and DIT. RMSD was computed for LAT1 α-carbons, substrates/inhibitor were excluded from RMSD calculation. **(C)** reports distances between Arg141 and substrates/inhibitor during transport. Dots indicate the frames identified as clusters centroids.

In these simulations, RMSF values computed for LAT1 α-carbons (Figure 4A) point out highly flexible regions, mainly corresponding to α-helices 1, 4, 6, 7, and 9. α-helices 11 and 12, required for LAT1 homo-dimerization (Napolitano et al., 2017a), are associated instead with very low local flexibility in T1 and D1 simulations, while α-helix 11 shows an increased flexibility in L1 simulation. As already mentioned above, the C-terminus shows the highest mobility.

Substrates/inhibitor path analysis does not provide any time-dependent information on transport; for this reason, the Z-axes distances between substrates/inhibitor and Arg141 were plotted for the tMD simulations. Arg141 was selected as reference, since it is placed in the middle of the transport channel, on α-helix 3, which presents the lowest RMSD between the starting OF and final IF structures. Arg141 itself is associated to the lowest RMSF value, suggesting that this residue encounters minor changes during OF → IF transitions and thus represents a good reference to trace substrates/inhibitor movements along the transport channel.

Comparing protein RMSD (Figure 4B) with Arg141 distance plots (Figure 4C), it appears that OF → IF LAT1 transition (between 2 and 4 ns) occurs before the transit (from 5 ns) of transported substrates/inhibitors close to the gate (Figures 3C). These data suggest that transported substrates can be delivered to the intracellular side only after the transporter structural rearrangement starts taking place. Summing-up, these tMD simulations, run in an artefactual setup (high spring constant), demonstrate a faster transport for substrates than for DIT. As shown in Figure 4C, Tyr and LME reach the binding site, near the Phe252 gate, approx. 1 ns before DIT, and remain there for 2 ns. Afterwards, transported substrates move down the second half of the transport channel, reaching the intracellular side in approximately 8 ns. Conversely, DIT reaches the binding site within 6 ns, stops there for about 2 ns, then exits very quickly from the transport channel. The distance profile analysis lets us hypothesize the existence of a second gate, other than Phe252, as described in AdiC (Krammer et al., 2016), which blocks the transport of the substrates until a suitable LAT1 rearrangement occurs.

In order to identify this putative gate, a cluster analysis was performed; results are reported in Table 2. Data discussed in detail in the text only refer to the centroids of the most populated clusters.

**Table 2:** tMD clusters for simulations with *k* = 100.

**tMD identifier**	**Number of clusters**	**Cluster ID**	**% Population**	**Substrates/inhibitor placing**
T1 (10 ns, *k* = 100)	13	C1	23	Channel
		C2	14.5	Extracellular side
		C3	10	Intracellular side
L1 (10 ns, *k* = 100)	20	C4	40	Extracellular side
		C5	26	Channel
		C6	20	Intracellular side
D1 (10 ns, *k* = 100)	13	C7	35.5	Extracellular side
		C8	28.5	Channel
		C9	15.5	Intracellular side

In all the 10 ns tMD simulations with *k* = 100, the three main clusters represent an initial, an intermediate and a final state of the transport, in which substrates and the inhibitor are at the extracellular side, buried in the channel and at the intracellular side of LAT1 channel.

**Figure 5:**
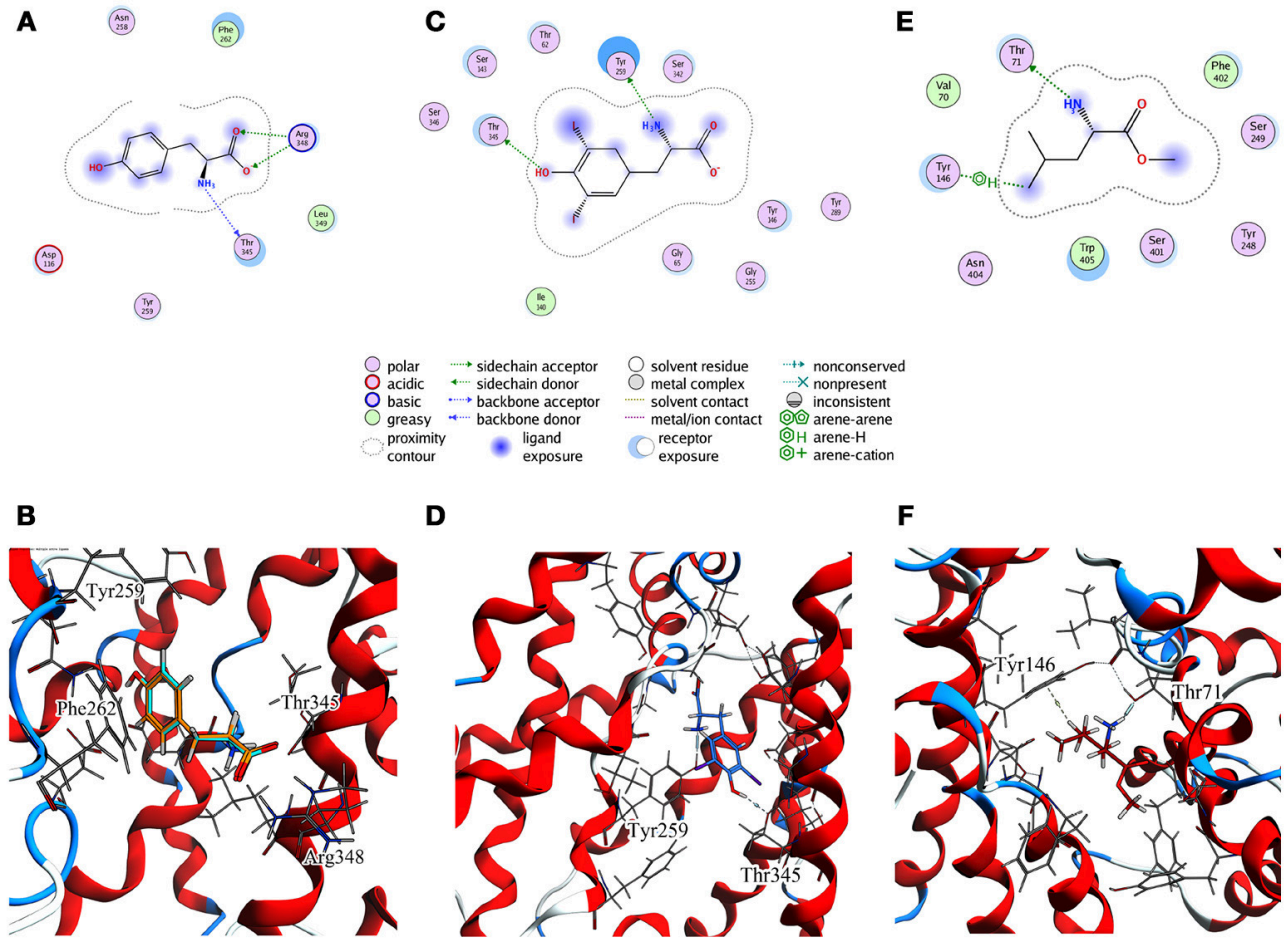
Cluster centroids for transported substrates and inhibitor. Tyr data in **(A,B)**, LME in **(C,D)**, DIT in **(E,F)**. **(A,C,E)** report the ligand interaction diagram of substrates with putative gating residues; **(B)** illustrates the conformation of C1-centroid and the superposition of C1 Tyr (orange) vs. Tyr molecular docking pose (light blue); **(D,F)** show DIT and LME placement in representative clusters. LAT1 is shown in ribbon representation. Tyr, LME, and DIT as well as LAT1 side chains are in stick representation.

For T1 the most populated cluster is C1; observing Tyr interactions with surrounding residues, it is possible to identify a putative inner gate, composed of three amino acids: Phe262, Arg348, and Thr345. Moreover, from IFFP, a total interaction energy (Shadnia et al., 2009) of −9.4 kcal/mol is evaluated (Table 3) and the relevant contribution from Phe262 Arg348, and Thr345 is confirmed. As shown in Figure 5A, hydrogen bonds are formed between the amino group of Tyr and the hydroxyl group of Thr345, and a salt bridge between the carboxylic group of Tyr and the basic group of Arg348.

**Table 3:** Interaction force fingerprint for simulations with *k* = 100.

**Cluster ID**	**Total interaction energy [kcal/mol]**	**Residues (Residue contribution [kcal/mol])**
C1	−9.4	Asp116 (−1.2), Tyr259 (−1.8), Phe262 (−2.6), Thr345 (−2.4), Arg348 (−2.7), Leu349 (−1.6)
C5	−12.0	Thr71 (−5.0), Ser143 (−1.7), Tyr147 (−7.6), Ser249 (−2.0), Phe252 (−2.1), Ala253 (−1.9), Phe402 (−2.9)
C8	−10.8	Tyr259 (−1.7) Phe262 (−2.0), Phe344 (−1.9), Thr345 (−2.9), Arg348 (−2.5)

To evaluate whether the interactions between Tyr and the putative inner gate residues pointed out by tMD simulation are relevant, we used molecular docking to sample Tyr binding modes more accurately, using the Tyr coordinates reported in C1 to address the binding site. We obtained five refined docking poses of Tyr, which had an RMSD ranging from 0.1 to 1.3 Å when compared to the Tyr binding mode observed in C1.

The top-scoring pose has an associated binding free energy of −7.4 kcal/mol and overlaps with the tMD reference pose with an RMSD value of 0.1 Å (Figure 5B), suggesting that C1 cluster is representative of an energetically-stable conformation of both substrate and transporter.

As already shown in our previous papers, a direct relationship exists between dissociation constant (*K*_*i*_) computed from binding free energy on molecular docking poses and *in vitro* data. Indeed, it has been reported that the accuracy of *in silico* estimated *K*_*i*_ values is approx. one order of magnitude with respect to the experimental values (Eberini et al., 2008; Galli et al., 2014), implying that the accuracy of the binding free energy is approximately 1.3 kcal/mol.

The contribution of each interacting residue to LAT1 model stability and to its affinity for the substrate Tyr has been estimated, with respect to the wild type, upon *in silico* cumulative mutations of each specified residue to alanine (Table 4). We found that the mutation of a single residue weakly affects the affinity for the transported substrate, whereas in cumulative mutations Arg348 seems to be the most relevant residue, among the investigated ones: the high ΔAffinity value suggests that this amino acid may play a pivotal role in the interaction with the substrate.

**Table 4:** *In silico* mutagenesis for C1 cluster. Mutations are sorted by differences in protein stability contribution.

**Mutations**	**ΔAffinity**	**ΔStability**
Y259Y, F262F, T345T, R348R	0.00	0.0
Y259Y, F262F, T345A, R348R	0.2	1.2
Y259A, F262F, T345T, R348R	0.1	1.5
Y259Y, F262F, T345T, R348A	0.4	1.6
Y259Y, F262A, T345T, R348R	0.2	2.0
Y259A, F262A, T345T, R348R	0.4	2.4
Y259A, F262A, T345A, R348R	0.5	2.5
Y259A, F262F, T345A, R348A	0.7	2.7
Y259Y, F262A, T345T, R348A	0.6	2.8
Y259Y, F262A, T345A, R348A	0.7	3.4
Y259A, F262A, T345T, R348A	0.6	3.5
Y259A, F262A, T345A, R348A	0.6	3.9

Only by mutating Phe262, ΔStability was appreciably affected, showing an increase with the number of cumulative mutations. When Phe262 and two or more other residues are simultaneously mutated, the ΔStability value is higher than 3 kcal/mol (Tokuriki et al., 2008).

These site-directed mutagenesis results, even if limited by their merely *in silico* nature, could be suggestive for further *in vitro* experiments, aimed at validating the proposed LAT1 transport mechanism.

The same analysis was repeated for both L1 (LME) and D1 (DIT) tMDs (Table 3). In C5, LME is placed in proximity of the outer gate where it establishes a Pi-stack interaction with Tyr147 and forms a H-bond with Thr71 (Figures 5C,D). The gating residue Phe252 also contributes to the total interaction energy. In C8, it was possible to identify crucial interactions between DIT, which is buried into the channel, and two residues, Thr345 and Tyr259; according to IFFP these two residues provide the highest contribution to the total interaction energy. Other residues, reported in Figures 5E,F, were recognized as important for the total interaction energy. C3, C6, and C9 clusters, representative of the final intracellular localization of transported substrates/inhibitor, and descriptive of the final states of dynamics, were not extensively analyzed since no interactions with LAT1 were observed.

In addition to the outer gate including Phe252, these tMD simulations suggest the presence of a second putative inner gate, located in the inward facing region of the transport channel; this involves Thr345, which interacts with Tyr and DIT. Through tMD it is possible to appreciate the different behavior of Tyr, LME and DIT since the substrates and the inhibitor transit through LAT1 with different timing.

### Decreasing spring constant tMD

Simulations of Tyr, LME, and DIT transport by LAT1 were repeated with *k* set at 90, 80, and 70 and results were processed through cluster analysis and IFFP as described above; an overview on the outcome is provided in Tables 5, 6.

**Table 5:** tMD clusters for simulations with *k* < 100.

**tMD identifier**	**Number of clusters**	**Cluster ID**	**% Population**	**Substrate/inhibitor placing**
T2 (5 ns, *k* = 90)	11	C10	68.4	Extracellular side
		C11	15	Channel
		C12	8	Intracellular side
T3 (5 ns, *k* = 80)	17	C13	57.6	Extracellular side
		C14	14.4	Channel
		C15	11.5	Channel
T4 (5 ns, *k* = 70)	8	C16	79.4	Extracellular side
		C17	10.4	Extracellular side
		C18	3.6	Channel
L2 (5 ns, *k* = 90)	6	C19	46	Extracellular side
		C20	20	Intracellular side
		C21	18	Channel
L3 (5 ns, *k* = 80)	16	C22	40	Channel
		C23	19	Channel
		C24	14	Extracellular side
L4 (5 ns, *k* = 70)	8	C25	64	Channel
		C26	20	Extracellular side
		C27	10	Extracellular side
D2 (5 ns, *k* = 90)	13	C28	55.2	Extracellular side
		C29	30.2	Channel
		C30	8	Channel
D3 (5 ns, *k* = 80)	3	C31	82	Extracellular side
		C32	17.8	Channel
4 (5 ns, *k* = 70)	5	C33	58.4	Extracellular side
		C34	22.8	Extracellular side
		C35	18.3	Extracellular side

**Table 6:** Interaction force fingerprint for simulations with *k* < 100.

**Cluster ID**	**Total interaction energy [kcal/mol]**	**Residues (Residue contribution [kcal/mol])**
C11	−13.3	Tyr259 (−1,7), Phe262 (−2.0), Phe344 (−1.9), Thr345 (−2.9), Arg348 (−2.5)
C14	−13.3	Tyr259 (−3.7), Thr345 (−1.4)
C15	−13.0	Gly114 (−1.0), Phe262 (−2.7), Arg348 (−3.6), Leu349 (−2.8), Val352 (−1.2)
C18	−9.0	Thr62 (−1.1), Ser143 (−2.2), Tyr146 (−1.8), Tyr259 (−6.6), Ser342 (−3.0), Thr345 (−1.4)
C21	−8.3	Phe262 (−1.6), Thr345 (−2.9), Arg348 (−1.7), Leu349 (−1.5)
C22	−16.6	Val70 (−2.0), Thr71 (−1.5), Tyr147 (−1.4), Ser249 (−1.6), Ser401 (−2.5), Asn404 (−1.2), Trp405 (−2.8)
C23	−10.9	Tyr259 (−4.2), Phe262 (−1.3), Thr345 (−1.8)
C25	−18.5	Gly67 (−1.9), Thr71 (−1.2), Ser249 (−1.6), Phe252 (−3.3), Ala253 (−1.8), Asn404 (−1.9), Trp405 (−2.6)
C29	−11.1	Ile140 (−1.1), Ser96 (−1.3), Tyr259 (−1.8), Gly294 (−1.7), Ser295 (−2.2)
C30	−11.4	Ile139 (−2.6), Ile140 (−4.7), Ser96 (−2.1), Asn211 (−1.8), Tyr259 (−1.5), Ser342 (−1.6)
C32	−19.4	Thr71 (−2.3), Gly74 (−1.8), Tyr146 (−2.7), Phe205 (−1.3), Ser354 (−1.3), Asn35 (−1.9), Trp358 (−2.2), Val36 (−1.1)

An extensive analysis of all the tMD trajectories and substrate paths (Supplementary Figure 5) shows that, as the spring constant *k* decreases, Tyr slips into LAT1 at different depths. Only in T2, Tyr completely crosses the membrane, exiting the LAT1 convex-hull. In T3, Tyr enters the transport channel, but does not exit the LAT1 convex-hull, whereas, in T4, Tyr is blocked just below the external gating region, Phe252. LAT1 rearrangements from OF to IF conformation occurring in T2, T3, T4 tMDs are shown in Supplementary Movies 2–4.

**Figure 6:**
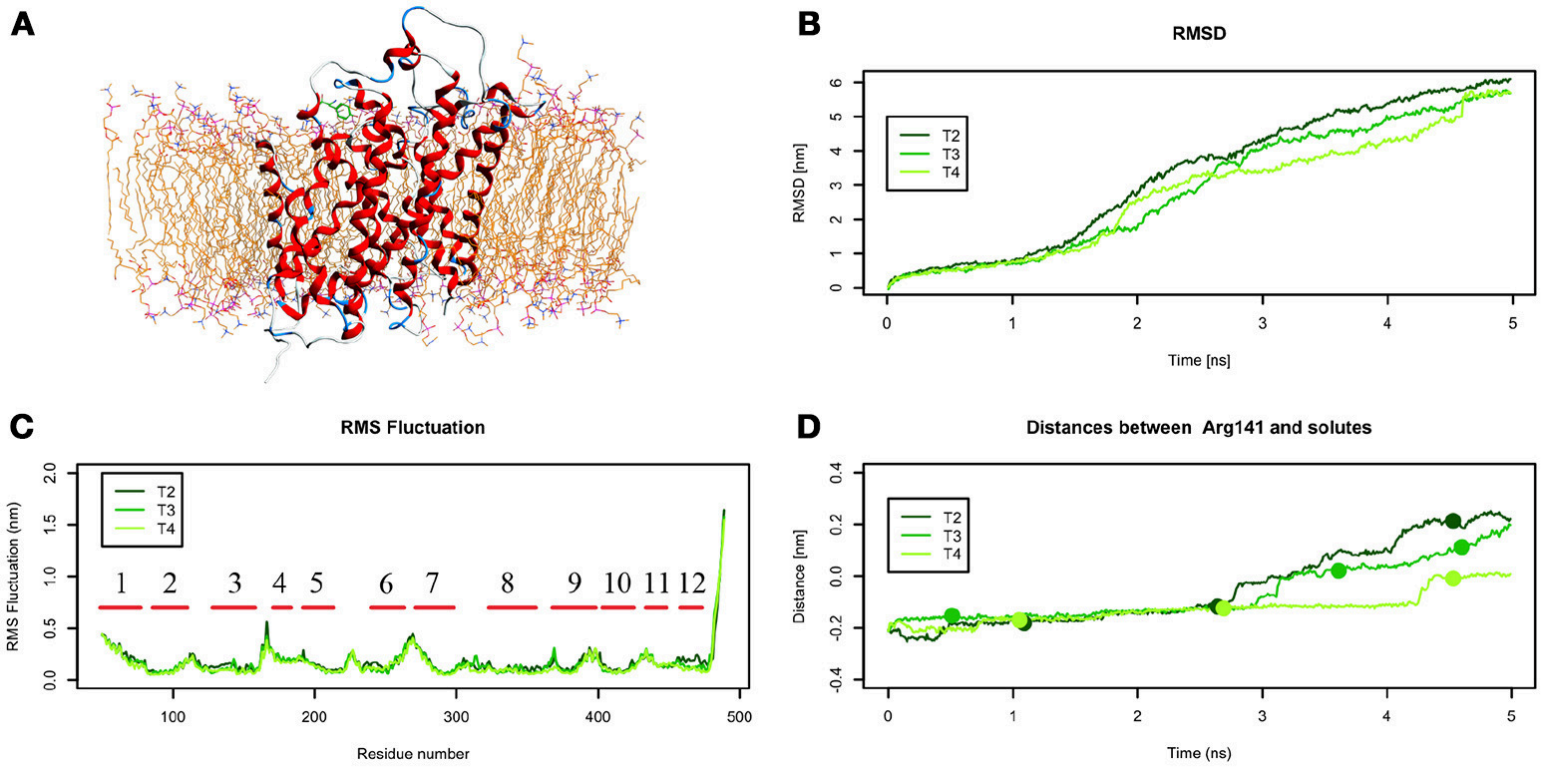
Geometrical changes of LAT1 during 5 ns tMD simulations of Tyr. **(A)** shows the starting position of Tyr in the tMD simulation with LAT1 OF structure into the POPC bilayer membrane. **(B)** shows RMSD profiles vs. time for LAT1 during OF → IF transitions. RMSD was computed for the α-carbons. **(C)** shows the RMSF for the three dynamics, computed for α-carbons. α-Helices are represented as red lines. **(D)** shows distances between Arg141 and substrates molecules. The points mark representative clusters.

The initial position of Tyr in the simulated system is shown in Figure 6.A. RMSD and RMSF plots (Figures 6B,C), for T2, T3 and T4 tMD simulations, do not significantly differ. The distance between Tyr and Arg141 was plotted as a function of time for all the 5ns tMDs, in order to assess the time spent by Tyr in the different regions of the LAT1 transport channel. In Figure 6.D it is possible to distinguish some steps in which Tyr is temporarily trapped, allowing us to hypothesize ongoing protein rearrangements. Cluster analysis of Tyr position along the LAT1 transport channel (Table 5) and IFFP assessment of residue contribution to interaction (Table 6) show that in C11 Tyr takes contact with the previously identified residues, i.e., Thr345, Arg348. Under the weaker spring constant value Tyr259 turns out to be a key residue in the putative inner gate. Other aromatic residues, Phe244 and Phe262, line the transport channel and participate in interactions with transported substrates. Also in this case, molecular docking simulations could reproduce the Tyr binding mode within this specific cluster (not shown). We obtained four refined docking poses of Tyr with an RMSD from the C11 Tyr reference coordinates ranging from 0.2 to 1.8 Å, and only one docking pose had an RMSD above the value of 2 Å, that is taken as a cut-off to discriminate between different binding modes (Poli et al., 2016). The top-scoring pose has a binding free energy of approx. −6.5 kcal/mol, associated with a RMSD of 0.2 Å, suggesting that the interaction mode between substrate and transporter pointed out by tMD simulation can also be carefully reproduced by molecular docking.

In C14, Tyr is close to Tyr259 and to Thr345, which are the main residues contributing to the IFFP. Interestingly, Phe252 is in an open conformation, whereas it is in a closed conformation in C15. The most populated cluster in T4 is representative of Tyr permanence in the extracellular side. C16 and C17 are representative of the Brownian movements of Tyr in the upper side, while C18 is representative of the last stage of the Tyr path; in this cluster, Tyr interacts with Thr345 and Tyr259. None of the 5 top-scoring poses obtained by docking simulation could reproduce the Tyr position in C18, suggesting that C18 is a transitory conformation of Tyr::LAT1 complex during the substrate transport.

**Figure 7:**
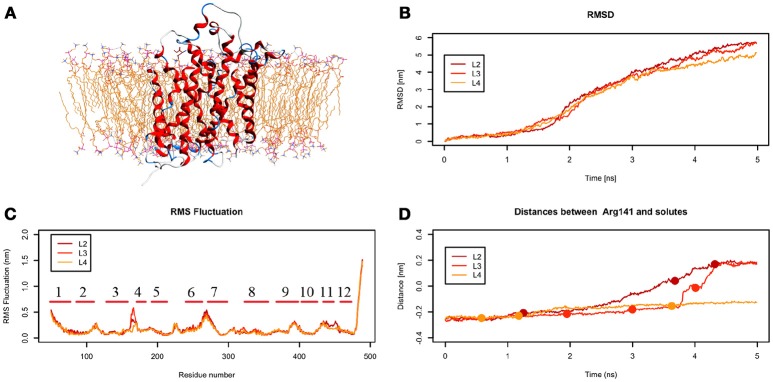
Geometrical changes of LAT1 during 5 ns tMD simulations of LME. **(A)** shows the starting position of LME in the tMD simulation with LAT1 OF structure into the POPC bilayer membrane. **(B)** shows RMSD profiles vs. time for LAT1 during OF → IF transitions. RMSD was computed for the α-carbons. **(C)** shows the RMSF for the three dynamics, computed for α-carbons. α-Helices are represented as red lines. **(D)** shows distances between Arg141 and LME. The points mark representative clusters.

From the analysis of the LME trajectories, the substrate reaches the intracellular space both in L2 and in L3. LME transport across LAT1 is shown in Supplementary Movies 5–7. The starting configuration of LME within the simulated system in tMDs is shown in Figure 7A. L2 and L3 have similar RMSD and RMSF profiles (Figures 7B,C), whereas in L4 LAT1 undergoes a less marked conformational change. With decreasing *k*, LME progresses with a linear path (L2) then takes a segmented track to eventually stop near the putative inner gate as also shown by the analysis of distances from Arg141 (Figure 7D). In C21 and C23 the substrate is near the putative inner gate, establishing interactions with Phe262 and Thr345. In C22 and C25, LME is near the outer gate; Thr71, Asn404 and Trp405 contribute significantly to the total interaction energy computed *via* IFFP. The remaining clusters, instead, are representative of Brownian motions.

**Figure 8:**
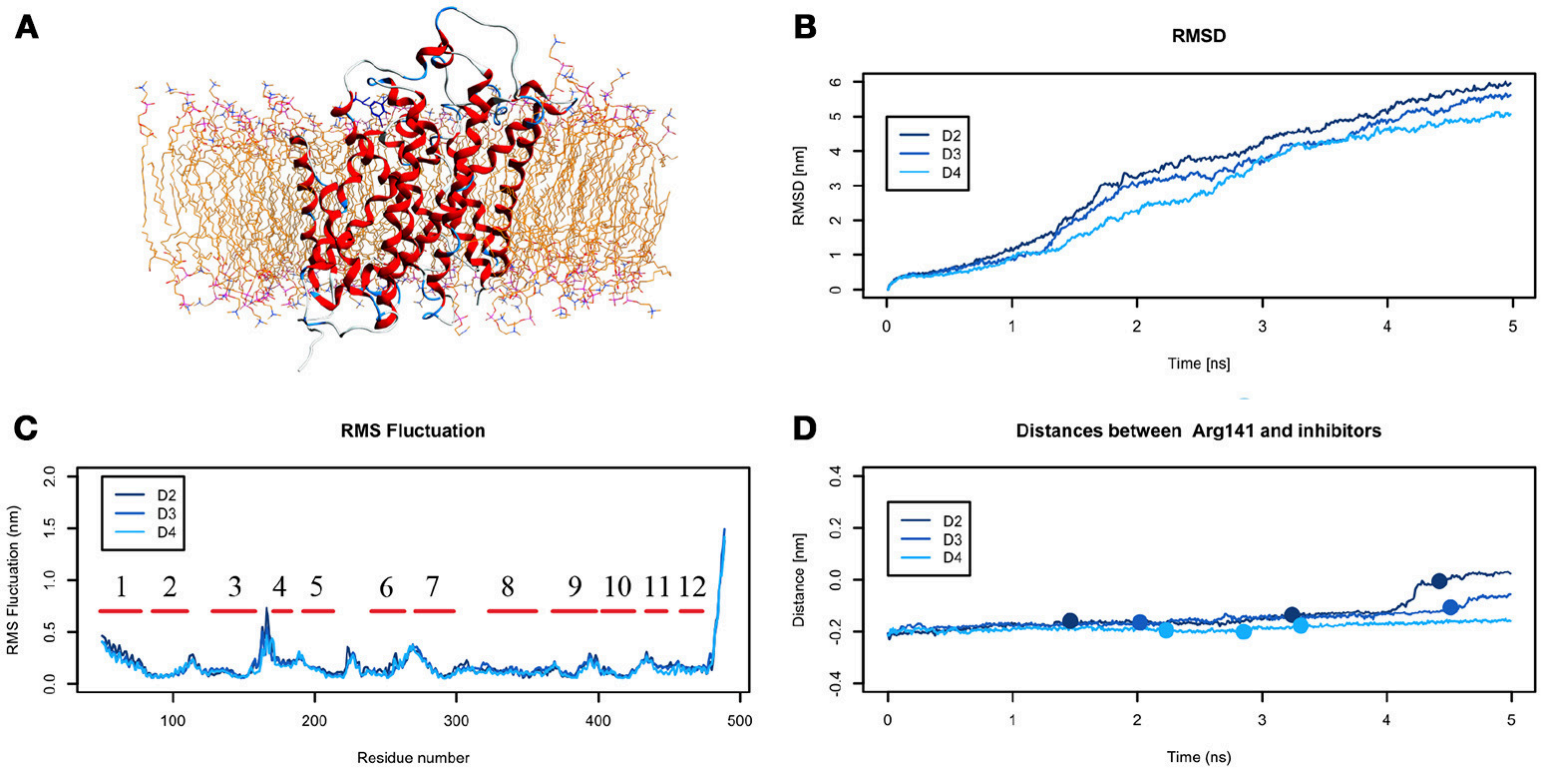
Geometrical changes of LAT1 during 5 ns tMD simulations of DITs. **(A)** shows the starting position of LME in the tMD simulation with LAT1 OF structure into the POPC bilayer membrane. **(B)** shows RMSD profiles vs. time for LAT1 during OF → IF transitions. RMSD was computed for the α-carbons. **(C)** shows the RMSF for three dynamics, computed for the α-carbons. α-helices are represented as red lines. **(D)** shows distances between Arg141 and DIT. The point mark the representative clusters.

The analysis of DIT path along LAT1 transport channel (Supplementary Figure 5), shows that the inhibitor neither reaches the intracellular side of the protein nor bypasses the outer gate residue Phe252. In fact, as the spring constant *k* decreases, DIT progressively fails to reach the deepest regions along the channel: (i) at *k* = 90 (D2) it is unable to cross the binding site, but bypasses the gate residue Phe252, (ii) at *k* = 80 (D3) it stops above the outer gate Phe252, and (iii) at *k* = 70 (D4) it remains in the extracellular domain, never reaching the outer gate. LAT1 rearrangements from OF to IF conformation occurring in D2, D3, D4 tMDs and DIT paths across LAT1 are shown in Supplementary Movies 8–10. The starting configuration of DIT in tMDs is shown in Figure 8A. The RMSD (Figure 8B) and RMSF profiles (Figure 8C) confirm the same general behavior previously described for other tMD simulations. In no case DIT passes through the channel, reaching the intracellular side, in agreement with its known inhibitory activity (Figure 8D). Varying the spring constant value *k*, DIT stops near Arg141 for approximately 1 ns (D2); then it remains in a putative outer binding site for approximately 0.5 ns (D3), and finally it stops in the extracellular side, never entering the transport channel of LAT1 for the whole simulated time (D4). From cluster analysis, in D2, a very populated cluster (C28, 93% of population) describes the DIT Brownian motions in the extracellular side. A second cluster, C29, describes the initial transport step, in which DIT enters the binding site, near the outer gate Phe252, and interacts with Ser401 and Lys77. Phe252 is exposed and in an open configuration. C31 shows Brownian motions of DIT in LAT1 extracellular side; C32 is representative of DIT motions at the entrance of the transport channel, where DIT interacts with Trp405 and Ser249. For D4, all clusters represent DIT Brownian motions in the extracellular side of the membrane.

Taken together, data from D2-4 tMD suggest that DIT inhibits LAT1 transport of amino acids with a competitive mechanism based on a molecular recognition that occurs outside the transport channel. Indeed, DIT may be recognized by LAT1 as a “fake substrate,” because of its similarity with the canonical transported substrates, but it can establish interactions only with LAT1 residues above Phe252 and external to the transport channel, preventing the interaction with, and the transport of, other substrates.

### Transport across LAT1

**Figure 9:**
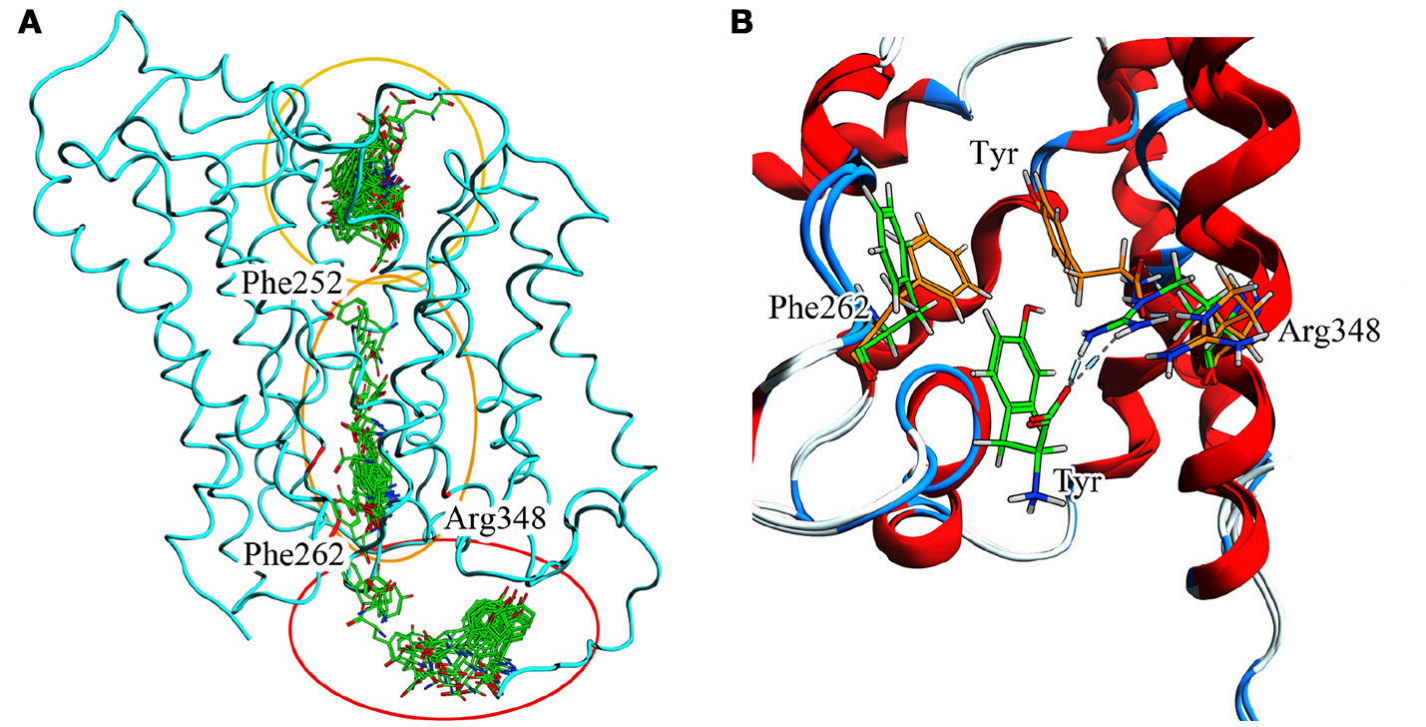
**(A)** Full T1 MD simulation. LAT1 is in an intermediate conformation, while Tyr is 100 ps frame skipped. Three different clusters can be observed: the first in the extracellular domain, near the inner gate (Phe252); the second in the transport channel, near Phe262; the third in the intracellular domain. These three clusters are representative of the LAT1 transport mechanism. **(B)** Inner gating mechanism. Phe262 and Arg348 torsion during channel exiting by Tyr. LAT1 is in ribbon representation; orange and green sticks correspond to different transport phases.

To summarize evidences collected *via* our *in silico* approach (Figure 9.A), it is possible to recognize Phe252 as the key gating residue in LAT1 outer region, as already described (Napolitano et al., 2017a); it is also possible to identify a more extended region around Phe262 that participates in the inner gating mechanism. This region includes also Phe244, Tyr259, Thr345, and Arg348. Phe262 closes the channel by steric hindrance, preventing substrate exit, in synergy with Arg348 (Figure 9B).

Ser342, already proposed as a residue involved in LAT1 binding (Geier et al., 2013), is crucial in substrate docking prior to translocation, as demonstrated both *in silico* and *in vitro* (Napolitano et al., 2017a).

To further support these findings, there is correspondence between Phe252 in LAT1 and the gating residue Trp202 in AdiC (Geier et al., 2013; Napolitano et al., 2017a). For other transporters of the APC family the transport mechanism is fully characterized: AdiC has a well-defined 2-gates mechanism (Trp202 and Trp293) (Krammer et al., 2016), whereas LeuT has a diffuse gating that involves more amino acids along the transport channel (Zhao and Noskov, 2013). On the other hand, Phe262 and the extended region around it, which could represent a second aromatic gate, suggest that LAT1 transport mechanism may be intermediate between those of AdiC and LeuT. In addition, Tyr tMD simulations show that Tyr259 is the residue contributing the largest energy to substrate interaction; this residue appears to have a role in stabilizing the substrate inside the channel.

Even though no experimental data on an inner gate are currently available for LAT1, our *in silico* results provide a first plausible description of its transport mechanism at an atomistic level, and can guide further *in vitro* experiments to confirm the role of the residues involved both in recognition and translocation of the transported substrates or inhibitors.

Finally, we analyzed the paths of water molecules across LAT1 channel through the whole simulation, for every tested condition. Overall, multiple inlet and outlet clusters appear near both channel entrances and the path analysis reveals that only few water molecules cross the entire channel. Instead, most paths link clusters on the same side of the protein and this is probably due to a gating mechanism that prevents the passage across the transport channel.

## Conclusions

With our research, based on single continuous tMD simulations, we propose, for the first time and at an atomistic level, the characterization of residues involved in LAT1 transport mechanism. The templates selected for modeling the OF and IF LAT1 states are the most suitable so far available, and have been extensively used in recent publications. Thanks to the application of different spring constant (*k*) values for each tested molecule, we identified a specific computational setup, through which we could investigate the transport mechanism of well-known transported substrates and inhibitor of LAT1. Even though the LAT1 OF → IF transition is forced by tMD simulations, DIT cannot pass through the LAT1 transport channel in specific scenarios which allow Tyr and LME to pass through. Specifically, decreasing *k* relaxed the transport mechanism, and enabled differential behaviors for transported substrates vs. inhibitor.

With the use of an evolving constraint, tMD does not sample an equilibrium ensemble, therefore not allowing to compute equilibrium properties, such as free energy profiles along the simulated processes. In addition, the presence of a constraint may force the system to overcome very high energy barriers. Despite these limitations, tMD is a very effective and acknowledged approach for investigating transition mechanisms (Ma et al., 2000; van der Vaart and Karplus, 2005; Ovchinnikov and Karplus, 2012). In this perspective, tMD overcomes classical MD methodologies, which are unable to capture large structural rearrangements for an all-atoms system over a suitable time scale. Our tMD simulations can discriminate between transported substrates and inhibitors, and can be proposed for developing novel inhibitors, useful for further pharmacological applications.

## Ethics statement

Authors are required to state the ethical considerations of their study in the manuscript, including for cases where the study was exempt from ethical approval procedures. Does the study presented in the manuscript involve human or animal subjects: No.

## Author contributions

LP set up the technique, performed part of the simulations, conducted part of the analyses and drafted the manuscript. CP conducted part of the analyses and revised the manuscript. TL and UG performed part of the simulations and contributed to the data analysis. CI and IE conceived the concept of this work. EG and IE supervised the work and revised the manuscript.

### Conflict of interest statement

The authors declare that the research was conducted in the absence of any commercial or financial relationships that could be construed as a potential conflict of interest.

## References

[B1] Augustyn E. Finke K. Zur A. A. Hansen L. Heeren N. Chien H. C.. (2016). LAT-1 activity of meta-substituted phenylalanine and tyrosine analogs. Bioorganic Med. Chem. Lett. 26, 2616–2621. 10.1016/j.bmcl.2016.04.023 27106710 PMC4875506

[B2] Boudker O. Verdon G. (2010). Structural perspectives on secondary active transporters. Trends Pharmacol. Sci. 31, 418–426. 10.1016/j.tips.2010.06.004 20655602 PMC2933288

[B3] Cheng M. H. Bahar I. (2013). Coupled global and local changes direct substrate translocation by neurotransmitter-sodium symporter ortholog LeuT. Biophys, J. 105, 630–639. 10.1016/j.bpj.2013.06.032 23931311 PMC3736663

[B4] Cheng M. H. Bahar I. (2014). Complete mapping of substrate translocation highlights the role of LeuT N-terminal segment in regulating transport cycle. PLoS Comput Biol. 10:e1003879. 10.1371/journal.pcbi.1003879 25299050 PMC4191883

[B5] Chiu M. Sabino C. Taurino G. Bianchi M. G. Andreoli R. Giuliani N.. (2017). GPNA inhibits the sodium-independent transport system l for neutral amino acids. Amino Acids 49, 1365–1372. 10.1007/s00726-017-2436-z 28516268

[B6] Colas C. Ung U. Schlessinger A. (2016). SLC transporters: structure, function and drug discovery. Medchemcomm 7, 1069–1081. 10.1039/C6MD00005C 27672436 PMC5034948

[B7] Corbeil C. R. Williams C. I. Labute P. (2012). Variability in docking success rates due to dataset preparation. J. Comput. Aided Mol. Des. 26, 775–786. 10.1007/s10822-012-9570-1 22566074 PMC3397132

[B8] Dehouck Y. Grosfils A. Folch B. Gilis D. Bogaerts P. Rooman M. (2009). Fast and accurate predictions of protein stability changes upon mutations using statistical potentials and neural networks: PoPMuSiC-2.0. Bioinformatics 25, 2537–2543. 10.1093/bioinformatics/btp445 19654118

[B9] Diallinas G. (2014). Understanding transporter specificity and the discrete appearance of channel-like gating domains in transporters. Front. Pharmacol. 5:207. 10.3389/fphar.2014.00207 25309439 PMC4162363

[B10] Dickens D. Webb S. D. Antonyuk S. Giannoudis A. Owen A. Rädisch S.. (2013). Transport of gabapentin by LAT1 (SLC7A5). Biochem. Pharmacol. 85, 1672–1683. 10.1016/j.bcp.2013.03.022 23567998

[B11] Eberini I. Rocco A. G. Mantegazza M. Gianazza E. Baroni A. Vilardo M. C.. (2008). Computational and experimental approaches assess the interactions between bovine beta-lactoglobulin and synthetic compounds of pharmacological interest. J. Mol. Graph. Model. 26, 1004–1013. 10.1016/j.jmgm.2007.08.006 17905618

[B12] Galli C. L. Sensi C. Fumagalli A. Parravicini C. Marinovich M. Eberini I. (2014). A computational approach to evaluate the androgenic affinity of iprodione, procymidone, vinclozolin and their metabolites. PLoS ONE 9:104822. 10.1371/journal.pone.0104822 25111804 PMC4128724

[B13] Gao X. Zhou L. Jiao X. Lu F. Yan C. Zeng X.. (2010). Mechanism of substrate recognition and transport by an amino acid antiporter. Nature 463, 828–832. 10.1038/nature08741 20090677

[B14] Geier E. G. Schlessinger A. Fan H. Gable J. E. Irwin J. J. Sali A.. (2013). Structure-based ligand discovery for the Large-neutral Amino Acid Transporter 1, LAT-1. Proc. Natl. Acad. Sci. U.S.A. 110, 5480–5485. 10.1073/pnas.1218165110 23509259 PMC3619328

[B15] Guerois R. Nielsen J. E. Serrano L. (2002). Predicting changes in the stability of proteins and protein complexes: a study of more than 1000 mutations. J. Mol. Biol. 320, 369–387. 10.1016/S0022-2836(02)00442-4 12079393

[B16] Haase C. Bergmann R. Fuechtner F. Hoepping A. Pietzsch J. (2007). L-type amino acid transporters LAT1 and LAT4 in cancer: uptake of 3-O-methyl-6-18F-fluoro-L-dopa in human adenocarcinoma and squamous cell carcinoma *in vitro* and *in vivo*. J. Nucl. Med. 48, 2063–2071. 10.2967/jnumed.107.043620 18056335

[B17] Humphrey W. Dalke A. Schulten K. (1996). VMD: visual molecular dynamics. J. Mol. Graph. 14, 33–38. 10.1016/0263-7855(96)00018-5 8744570

[B19] Ilgü H. Jeckelmann J.-M. Gapsys V. Ucurum Z. de Groot B. L. Fotiadis D. (2016). Insights into the molecular basis for substrate binding and specificity of the wild-type L-arginine/agmatine antiporter AdiC. *Proc. Natl. Acad. Sci*. U.S.A. 113, 10358–10363. 10.1073/pnas.1605442113 PMC5027449 27582465

[B18] Ilgü H. Jeckelmann J. M. Colas C. Ucurum Z. Schlessinger A. Fotiadis D. (2018). Effects of mutations and ligands on the thermostability of the L-arginine/agmatine antiporter adic and deduced insights into ligand-binding of human L-type amino acid transporters. Int. J. Mol. Sci. 19:E918. 10.3390/ijms19030918 29558430 PMC5877779

[B20] Isberg V. Graaf C. De Bortolato A. Cherezov V. Katritch V. Gloriam D. E. (2016). Generic GPCR residue numbers - aligning topology maps minding the gaps. HHS Public Access. 36, 22–31. 10.1016/j.tips.2014.11.001 25541108 PMC4408928

[B21] Jurik A. Zdrazil B. Holy M. Stockner T. Sitte H. H. Ecker G. F. (2015). A binding mode hypothesis of tiagabine confirms liothyronine effect on γ-aminobutyric acid transporter 1 (GAT1). J. Med. Chem. 58, 2149–2158. 10.1021/jm5015428 25679268 PMC4360375

[B22] Kazmier K. Claxton D. P. Mchaourab H. S. (2017). Alternating access mechanisms of LeuT-fold transporters: trailblazing towards the promised energy landscapes. Curr. Opin. Struct. Biol. 45, 100–108. 10.1016/j.sbi.2016.12.006 28040635 PMC5491374

[B23] Koshy C. Schweikhard E. S. Gärtner R. M. Perez C. Yildiz Ö. Ziegler C. (2013). Structural evidence for functional lipid interactions in the betaine transporter BetP. EMBO J. 32, 3096–3105. 10.1038/emboj.2013.226 24141878 PMC3844952

[B24] Kowalczyk L. Ratera M. Paladino A. Bartoccioni P. Errasti-Murugarren E. Valencia E. (2011). Molecular basis of substrate-induced permeation by an amino acid antiporter. *Proc Natl Acad Sci*. U.S.A. 108, 3935–3940. 10.1073/pnas.1018081108 PMC3054010 21368142

[B25] Krammer E. M. Ghaddar K. Andre B. Provost M. (2016). Unveiling the mechanism of arginine transport through AdiC with molecular dynamics simulations: the guiding role of aromatic residues. PLoS ONE 11:160219. 10.1371/journal.pone.0160219 27482712 PMC4970712

[B26] Kroncke B. M. Horanyi P. S. Columbus L. (2010). Structural origins of nitroxide side chain dynamics on membrane protein alpha-helical sites. Biochemistry 49, 10045–10060. 10.1021/bi101148w 20964375 PMC2991438

[B27] Labute P. (2008). The generalized Born/volume integral implicit solvent model: estimation of the free energy of hydration using London dispersion instead of atomic surface area. J. Comput. Chem. 29, 1693–1698. 10.1002/jcc.20933 18307169

[B28] Lee J. Sands Z. A. Biggin P. C. (2016). A numbering system for MFS transporter proteins. Front. Mol. Biosci. 3, 1–13. 10.3389/fmolb.2016.00021 27314000 PMC4889909

[B29] Li W. Cowley A. Uludag M. Gur T. McWilliam H. Squizzato S.. (2015). The EMBL-EBI bioinformatics web and programmatic tools framework. Nucleic Acids Res. 43, W580–W584. 10.1093/nar/gkv279 25845596 PMC4489272

[B30] Lomize M. A. Pogozheva I. D. Joo H. Mosberg H. I. Lomize A. L. (2012). OPM database and PPM web server: resources for positioning of proteins in membranes. Nucleic Acids Res. 40:D370-6. 10.1093/nar/gkr703 21890895 PMC3245162

[B31] Ma D. Lu P. Yan C. Fan C. Yin P. Wang J.. (2012). Structure and mechanism of a glutamate-GABA antiporter. Nature 483, 632–636. 10.1038/nature10917 22407317

[B32] Ma J. Sigler P. B. Xu Z. Karplus M. (2000). A dynamic model for the allosteric mechanism of GroEL. J. Mol. Biol. 302, 303–313. 10.1006/jmbi.2000.4014 10970735

[B33] Naïm M. Bhat S. Rankin K. N. Dennis S. Chowdhury S. F. Siddiqi I.. (2007). Solvated Interaction Energy (SIE) for scoring protein-ligand binding affinities. 1. Exploring the parameter space. J. Chem. Inf. Model. 47, 122–133. 10.1021/ci600406v 17238257

[B34] Napolitano L. Galluccio M. Scalise M. Parravicini C. Palazzolo L. Eberini I.. (2017a). Novel insights into the transport mechanism of the human amino acid transporter LAT1 (SLC7A5). Probing critical residues for substrate translocation. Biochim. Biophys. Acta Gen. Subj. 1861, 727–736. 10.1016/j.bbagen.2017.01.013 28088504

[B35] Napolitano L. Scalise M. Koyioni M. Koutentis P. Catto M. Eberini I. (2017b). Potent inhibitors of human LAT1 (SLC7A5) transporter based on dithiazole and dithiazine compounds for development of anticancer drugs. Biochem. Pharmacol. 1, 1–14. 10.1016/j.bcp.2017.07.006 28709952

[B36] Ovchinnikov V. Karplus M. (2012). Analysis and elimination of a bias in targeted molecular dynamics simulations of conformational transitions: application to calmodulin. J. Phys. Chem. B. 116, 8584–8603. 10.1021/jp212634z 22409258 PMC3406239

[B37] Perez C. Koshy C. Ressl S. Nicklisch S. Krämer R. Ziegler C. (2011). Substrate specificity and ion coupling in the Na^+^/betaine symporter BetP. EMBO J. 30, 1221–1229. 10.1038/emboj.2011.46 21364531 PMC3094121

[B38] Phillips J. C. Braun R. Wang W. Gumbart J. Tajkhorshid E. Villa E.. (2005). Scalable molecular dynamics with NAMD. J. Computat. Chem. 26, 1781–802. 10.1002/jcc.20289 16222654 PMC2486339

[B39] Platonova N. Parravicini C. Sensi C. Paoli A. Colombo M. Neri A.. (2017). Identification of small molecules uncoupling the Notch::Jagged interaction through an integrated high-throughput screening. PLoS ONE 12:182640. 10.1371/journal.pone.0182640 29099834 PMC5669421

[B40] Poli G. Martinelli A. Tuccinardi T. (2016). Reliability analysis and optimization of the consensus docking approach for the development of virtual screening studies. J. Enzyme Inhib. Med. Chem. 31, 167–173. 10.1080/14756366.2016.1193736 27311630

[B41] Quick M. Winther A-ML Shi L. Nissen P. Weinstein H. Javitch J. A. (2009). Binding of an octylglucoside detergent molecule in the second substrate (S2) site of LeuT establishes an inhibitor-bound conformation. *Proc. Natl. Acad. Sci*. U.S.A. 106, 5563–5568. 10.1073/pnas.0811322106 PMC2667088 19307590

[B42] Ressl S. Terwisscha Van Scheltinga A. C. Vonrhein C. Ott V. Ziegler C. (2009). Molecular basis of transport and regulation in the Na^+^/betaine symporter BetP. Nature 458, 47–52. 10.1038/nature07819 19262666

[B43] Scalise M. Galluccio M. Console L. Pochini L. Indiveri C. (2018). The human SLC7A5 (LAT1): the intriguing histidine/large neutral amino acid transporter and its relevance to human health. Front. Chem. 6:243. 10.3389/fchem.2018.00243 29988369 PMC6023973

[B44] Schlessinger A. Khuri N. Giacomini K. M. Sali A. (2013). Molecular modeling and ligand docking for solute carrier (SLC) transporters. Curr. Top. Med. Chem. 13, 843–856. 10.2174/1568026611313070007 23578028 PMC4056341

[B45] Schlitter J. Engels M. Krüger P. (1994). Targeted molecular dynamics: a new approach for searching pathways of conformational transitions. J. Mol. Graph. 12, 84–89. 10.1016/0263-7855(94)80072-3 7918256

[B46] Sensi C. Daniele S. Parravicini C. Zappelli E. Russo V. Trincavelli M. L.. (2014). Oxysterols act as promiscuous ligands of class-A GPCRs: *in silico* molecular modeling and *in vitro* validation. Cell Signal. 26, 2614–2620. 10.1016/j.cellsig.2014.08.003 25152366

[B47] Shadnia H. Wright J. S. Anderson J. M. (2009). Interaction force diagrams: new insight into ligand-receptor binding. J. Comput. Aided Mol. Des. 23, 185–194. 10.1007/s10822-008-9250-3 18989626

[B48] Shaffer P. L. Goehring A. Shankaranarayanan A. Goaux E. (2009). Structure and mechanism of a Na^+^ independent amino acid transporter. Science 325, 1010–1014. 10.1126/science.1176088 19608859 PMC2851542

[B49] Shaikh S. A. Tajkhorshid E. (2010). Modeling and dynamics of the inward-facing state of a Na^+^/Cl^−^ dependent neurotransmitter transporter homologue. PLoS Comput Biol. 6:1000905. 10.1371/journal.pcbi.1000905 20865057 PMC2928745

[B50] Shimamura T. Weyand S. Beckstein O. Rutherford N. G. Hadden J. M. Sharpies D.. (2010). Molecular basis of alternating access membrane transport by the sodium-hydantoin transporter Mhp1. Science 328, 470–473. 10.1126/science.1186303 20413494 PMC2885435

[B51] Simmons K. J. Jackson S. M. Brueckner F. Patching S. G. Beckstein O. Ivanova E. (2014). Molecular mechanism of ligand recognition by membrane transport protein, Mhp1. EMBO J. 21, 11–27. 10.15252/embj.201387557 PMC4195764 24952894

[B52] Singh S. K. Piscitelli C. L. Yamashita A. Gouaux E. (2008). A competitive inhibitor traps LeuT in an open-to-out conformation. Science 322, 1655–1661. 10.1126/science.1166777 19074341 PMC2832577

[B53] Stolzenberg S. Quick M. Zhao C. Gotfryd K. Khelashvili G. Gether U.. (2015). Mechanism of the association between Na^+^ binding and conformations at the intracellular gate in neurotransmitter:sodium symporters. J. Biol. Chem. 290, 13992–14003. 10.1074/jbc.M114.625343 25869126 PMC4447972

[B54] Tărlungeanu D. C. Deliu E. Dotter C. P. Kara M. Janiesch P. C. Scalise M.. (2016). Impaired amino acid transport at the blood brain barrier is a cause of autism spectrum disorder. Cell 167, 1481–1494.e18. 10.1016/j.cell.2016.11.013 27912058 PMC5554935

[B55] Tokuriki N. Stricher F. Serrano L. Tawfik D. S. (2008). How protein stability and new functions trade off. PLoS Comput. Biol. 4, 35–37. 10.1371/journal.pcbi.1000002 18463696 PMC2265470

[B56] van der Vaart A. Karplus M. (2005). Simulation of conformational transitions by the restricted perturbation-targeted molecular dynamics method. J. Chem. Phys. 122:114903. 10.1063/1.1861885 15836253

[B57] Weyand S. Shimamura T. Yajima S. Suzuki S. N. I. Mirza O. Krusong K.. (2008). Structure and molecular mechanism of a nucleobase-cation-symport-1 family transporter. Science 322, 709–713. 10.1126/science.1164440 18927357 PMC2885439

[B58] Zhao C. Noskov S. Y. (2013). The molecular mechanism of ion-dependent gating in secondary transporters. PLoS Comput Biol. 9:1003296. 10.1371/journal.pcbi.1003296 24204233 PMC3812048

[B59] Zhou Z. Zhen J. Karpowich N. K. Law C. J. Reith M. E. A. Wang D. N. (2009). Antidepressant specificity of serotonin transporter suggested by three LeuT-SSRI structures. Nat. Struct. Mol. Biol. 16, 652–657. 10.1038/nsmb.1602 19430461 PMC2758934

[B60] Zur A. A. Chien H. C. Augustyn E. Flint A. Heeren N. Finke K.. (2016). LAT1 activity of carboxylic acid bioisosteres: evaluation of hydroxamic acids as substrates. Bioorgan. Med. Chem. Lett. 26, 5000–5006. 10.1016/j.bmcl.2016.09.001 27624080 PMC5076878

