# Supplementary material for: In silico Description of LAT1 Transport Mechanism at an Atomistic Level
Source: Front Chem. 2018 Aug 24;6:350. doi: 10.3389/fchem.2018.00350 (PMC6117385; doi:10.3389/fchem.2018.00350)
Supplement: Supplementary file 11 [file Data_Sheet_1.PDF]

```
AdiC    1   -----------------------------------------MSSDADAHKVGLIPVTLMV   19
GadC    1   ----------------------------------------MATSVQTGKAKQLTLLGFFA   20
LAT1    1   MAGAGPKRRALAAPAAEEKEEAREKMLAAKSADGSAPAGEGEG-VTLQRNITLLNGVAII   59

AdiC   20   SGNIMGSGVFLLPANLASTGG---IAIYGWLVTIIGALGLSMVYAKMSFLD-PSPGGSYA   75
GadC   21   ITASMVMAVYEYPTFATSGFS---LVFFLLLGGILWFIPVGLCAAEMATVDGWEEGGVFA   77
LAT1   60   VGTIIGSGIFVTPTGVLKEAGSPGLALVVWAACGVFSIVGALCYAELGTTI-SKSGGDYA   118

AdiC   76   YARRCFGPFLGYQTNVLYWLACWIGNIAMVVIGVG--YLSYFFPILKDP-LVLTITCVVV   133
GadC   78   WVSNTLGPRWGFAAISFGYLQIAIGFIPMLYFVLGALSYILKWPALNEDPITKTIAALII   137
LAT1  119   YMLEVYGSLPAFLKLWIELLIIRPSSQYIVALVFATYLLKPLFPTCPVPEEAAKLVACLC   178

AdiC  134   LWIFVLLNIVGPKMITRVQAVATVLALIPIVGIAVFGWFWFRGETYMAA-----WNVSGL   186
GadC  138   LWALALTQFGGTKYTARIAKVGFFAGILLPAFILIALAAIYLHSGAPVAIEMDSKTFFPD   197
LAT1  179   VLLLTAVNCYSVKAATRVQDAFAAAKLLALALIILLGFVQIGKGDVSNLDPNFSFEG-TK   237

AdiC  187   GTFGAIQSTLNVTLWSFIGVESASVAAGVVKNPKRNVPIATIGGVLIAAVCYVLSTTAIM   247
GadC  198   FSKVGTLVVFVAFILSYMGVEASATHVNEMSNPGRDYPLAMLLLMVAAICLSSVGGLSIA   257
LAT1  138   LDVGNIVLALYSGLFAYGGWNYLNFVTEEMINPYRNLPLAIIISLPIVTLVYVLTNLAYF   297

AdiC  248   GMIPNAALRVSASPFGDAARMALGD-----TAGAIVSFCAAAGCLGSLGGWTLLAGQTAK   302
GadC  258   MVIPGNEINLSAGVMQTFTVLMSHVAPEIEWTVRVISALLLLGVLAEIASWIVGPSRGMY   317
LAT1  298   TTLSTEQMLSSEAVAVDFGNYHLG------VMSWIIPVFVGLSCFGSVNGSLFTSSRLFF   351

AdiC  303   AAADDGL-FPPIFARVNKAGTPVAGLIIVGILMT----IFQLSSISPNATKEFGLVSSVS   357
GadC  318   VTAQKNL-LPAAFAKMNKNGVPVTLVISQLVITSIALIILTNTGGGNNMSFLIALALTVV   376
LAT1  352   VGSREGH-LPSILSMIHPQLLTPVPSLVFTCVMT---LLYAFSKDIFSVINFFSFFNWLC   407

AdiC  358   VIFTLVPYLYTCAALLLLGHGH---------FGKARPAYLAVTTIAFLYCIWAVVGSGA-   407
GadC  377   IYLCAYFMLFIGYIVLVLKHPDLKRTFNIPGGKGVKLVVAIVGLLTSIMAFIVSFLPPDN   436
LAT1  408   VALAIIGMIWLRHRKPELER-----------PIKVNLALPVFFILACLFLIAVSFWKTP-   455

AdiC  408   ---------KEVMWSFVTLMVITAMYALNYNRLHKNPYPLDAPISKD-------------   445
GadC  437   IQGDSTDMYVELLVVSFLVVLALPFILYAVHDRKGKANTGVTLEPINSQNAPKGHFFLHP   496
LAT1  456   ---------VECGIGFTIILSGLPVYFFGVWWKNKPKWLLQGIFSTTVLCQKLMQVVPQE   506

AdiC    -   ---------------                                                -
GadC  497   RARSPHYIVMNDKKH                                                511
LAT1  507   T--------------                                                507
```

Multiple sequence alignment. Primary structures of homologous APC transporters are aligned according to a previously published multiple alignments (Napolitano, Galluccio, et al. 2017; Napolitano, Scalise, et al. 2017).
