# Supplementary material for: In silico Description of LAT1 Transport Mechanism at an Atomistic Level
Source: Front Chem. 2018 Aug 24;6:350. doi: 10.3389/fchem.2018.00350 (PMC6117385; doi:10.3389/fchem.2018.00350)
Supplement: Supplementary file 12 [file Data_Sheet_2.PDF]

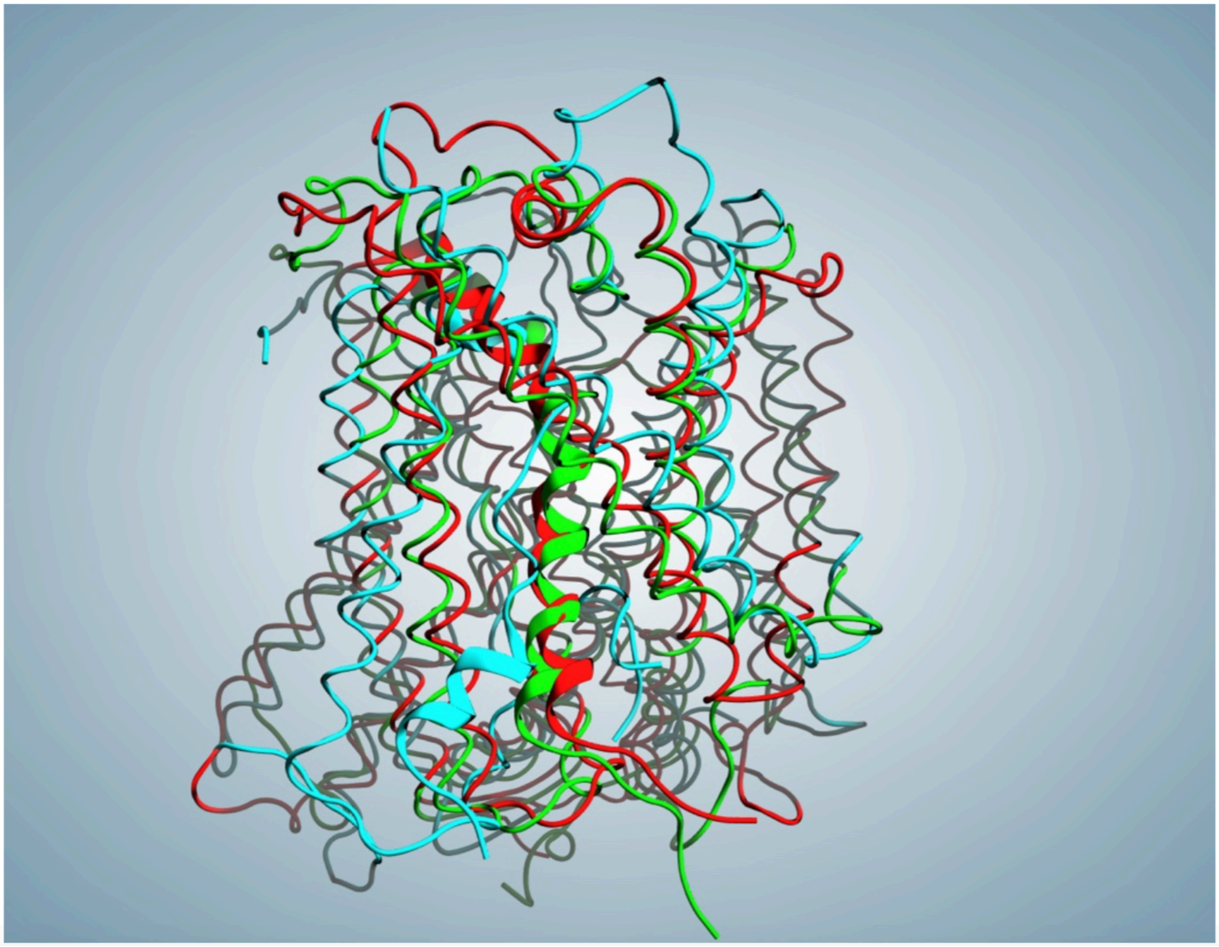

Supplementary Figure 1: Superposition of APC transporter structures. The structures of three LAT1 homologous proteins are superposed by structural alignment. AdiC, ApcT and GadC are represented as red, green and cyan lines, respectively. For each protein, Helix 1, which is connected with the largest conformational changes in the transition from the OF towards the IF state, is shown as ribbons.

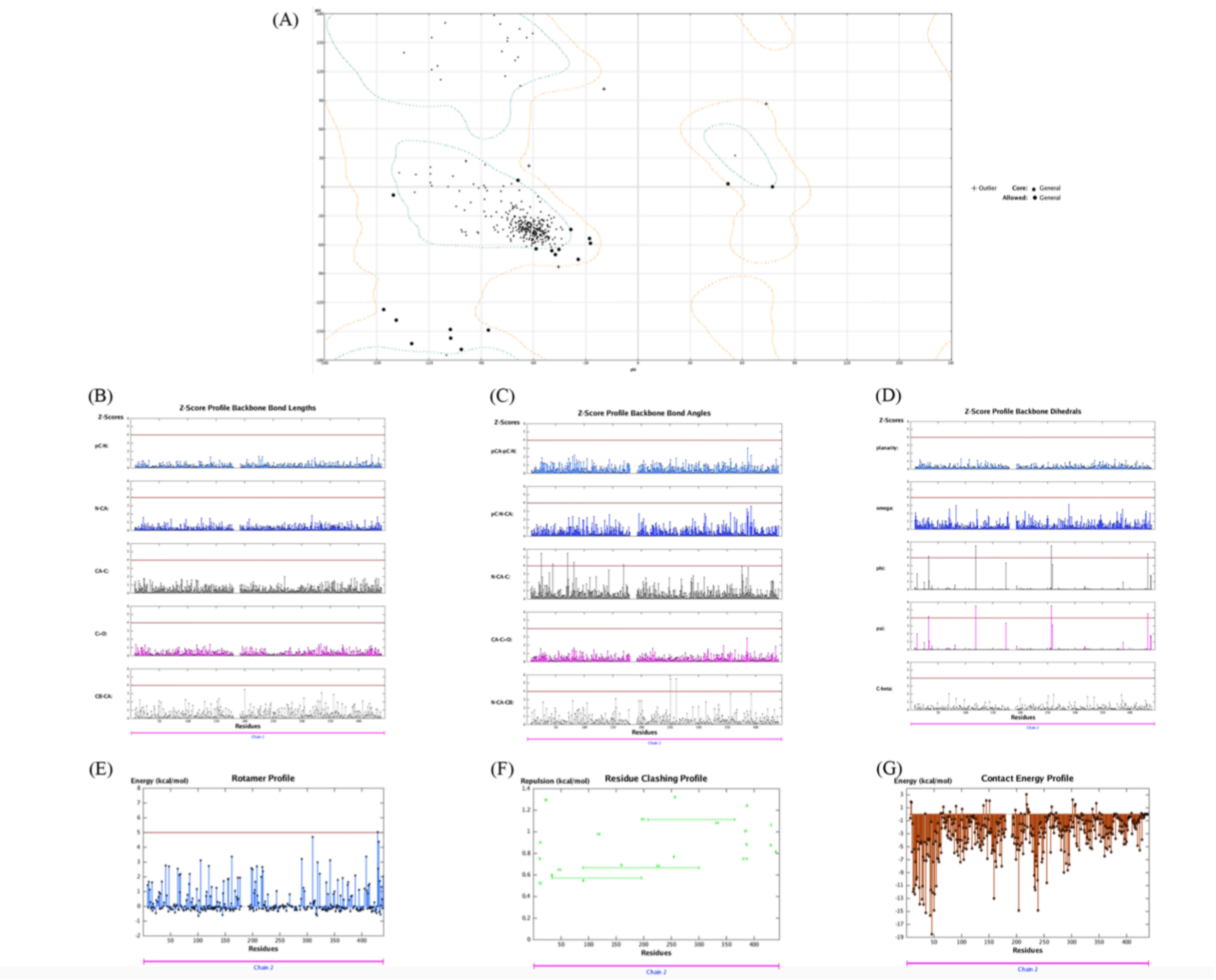

Supplementary Figure 2a: Protein geometry quality validation: (A) Ramachandran plot, (A) bond lengths, (C) bond angles, (D) dihedrals, (E) rotamers, (F) atom clashes, and (G) contact energies for AdiC structure, used as template for modelling OF LAT1 model.

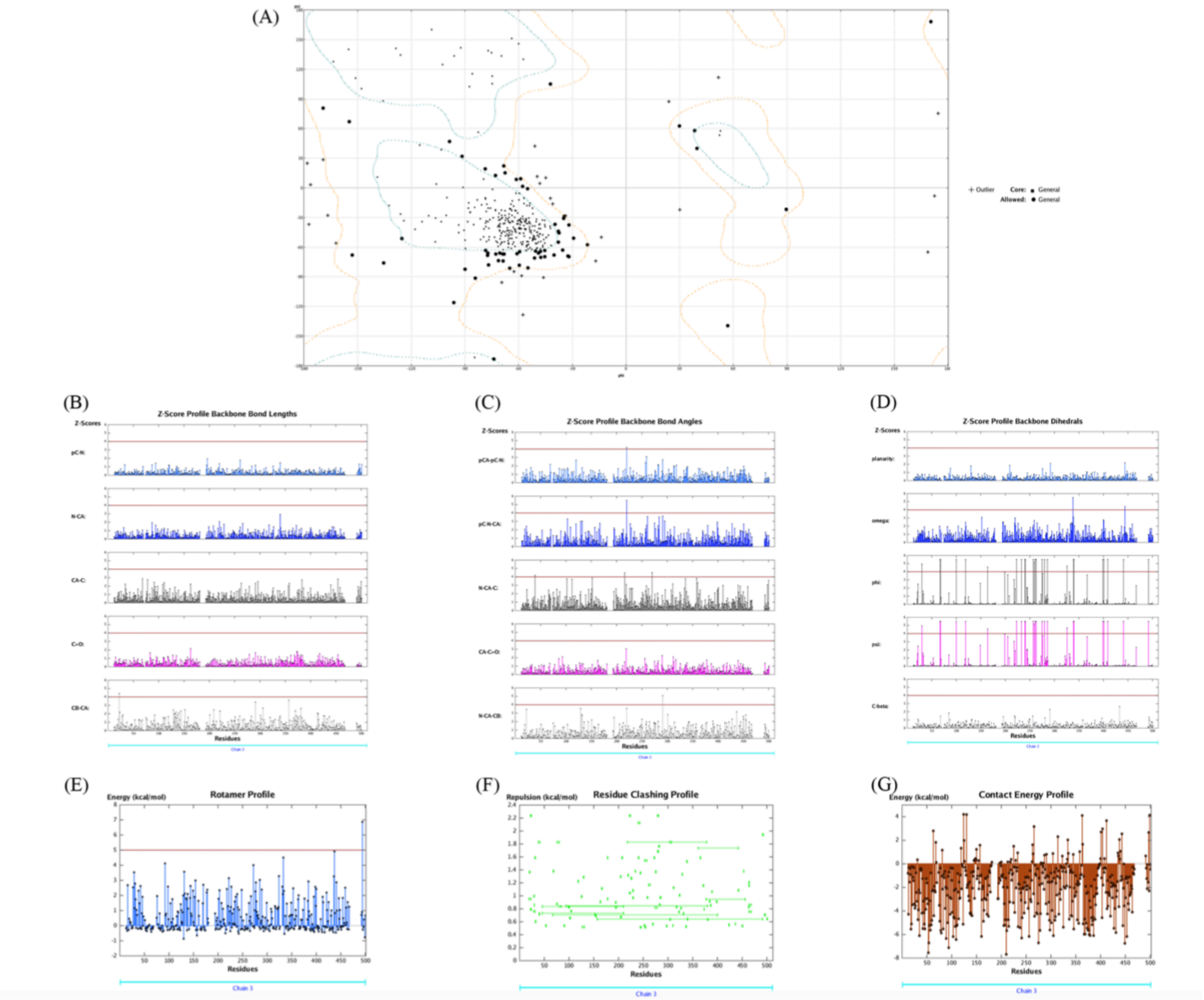

Supplementary Figure 2b: Protein geometry quality validation: Protein geometry quality validation: (A) Ramachandran plot, (A) bond lengths, (C) bond angles, (D) dihedrals, (E) rotamers, (F) atom clashes, and (G) contact energies for GadC structure, used as template for modelling IF LAT1 model.

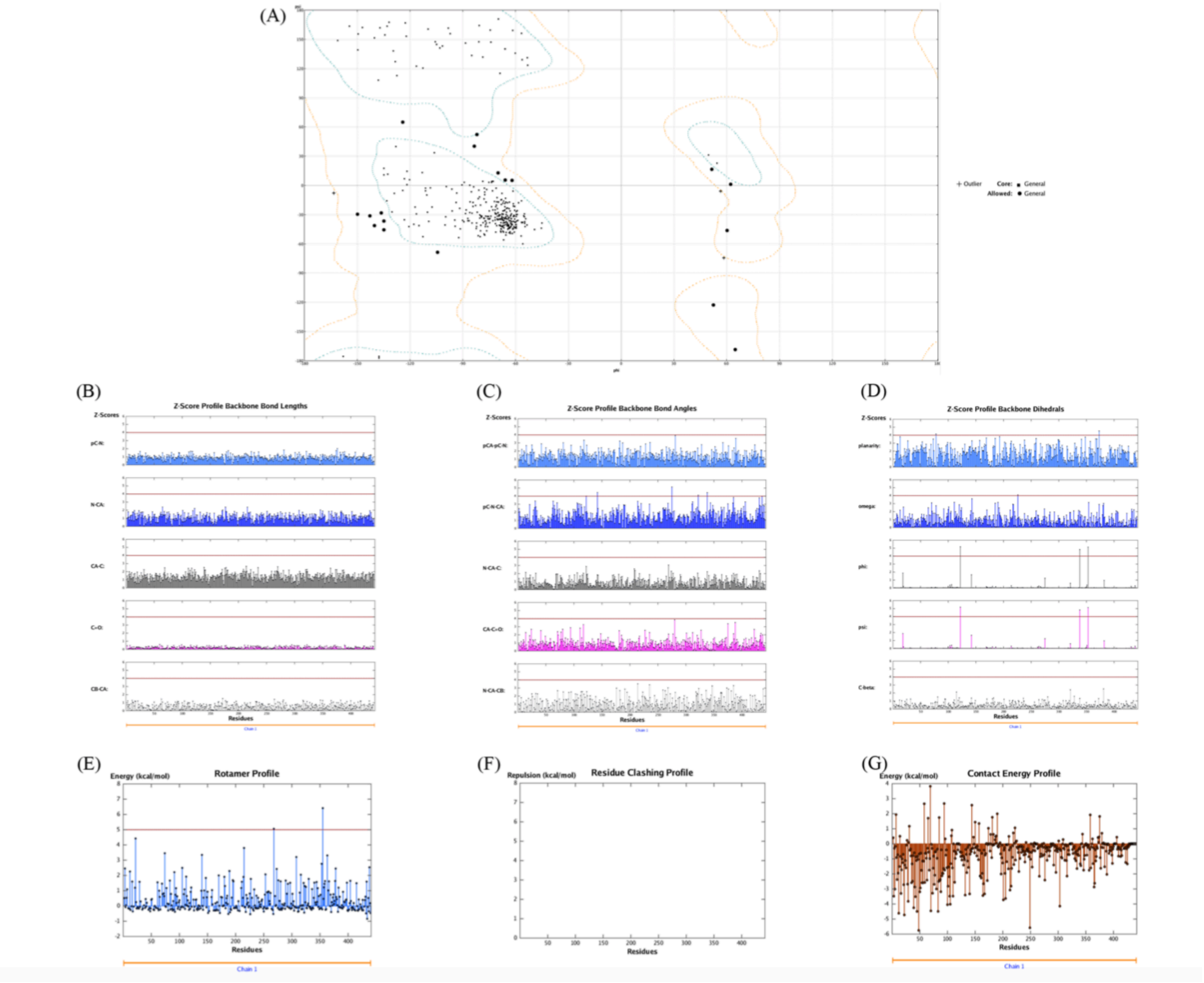

Supplementary Figure 2c: Protein geometry quality validation: Protein geometry quality validation: (A) Ramachandran plot, (A) bond lengths, (C) bond angles, (D) dihedrals, (E) rotamers, (F) atom clashes, and (G) contact energies for OF LAT1 model.

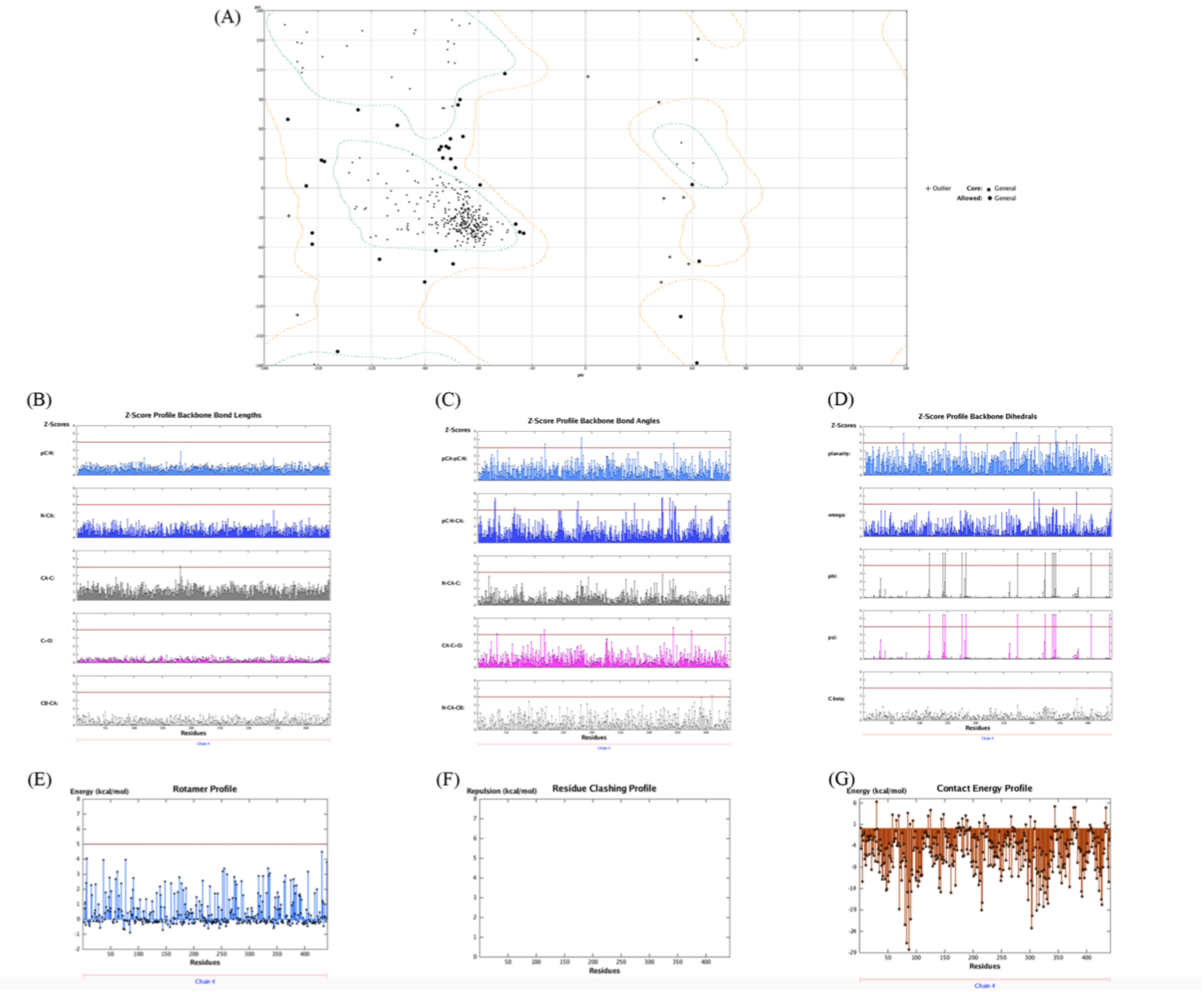

Supplementary Figure 2d: Protein geometry quality validation: Protein geometry quality validation: (A) Ramachandran plot, (A) bond lengths, (C) bond angles, (D) dihedrals, (E) rotamers, (F) atom clashes, and (G) contact energies for IF LAT1 model.

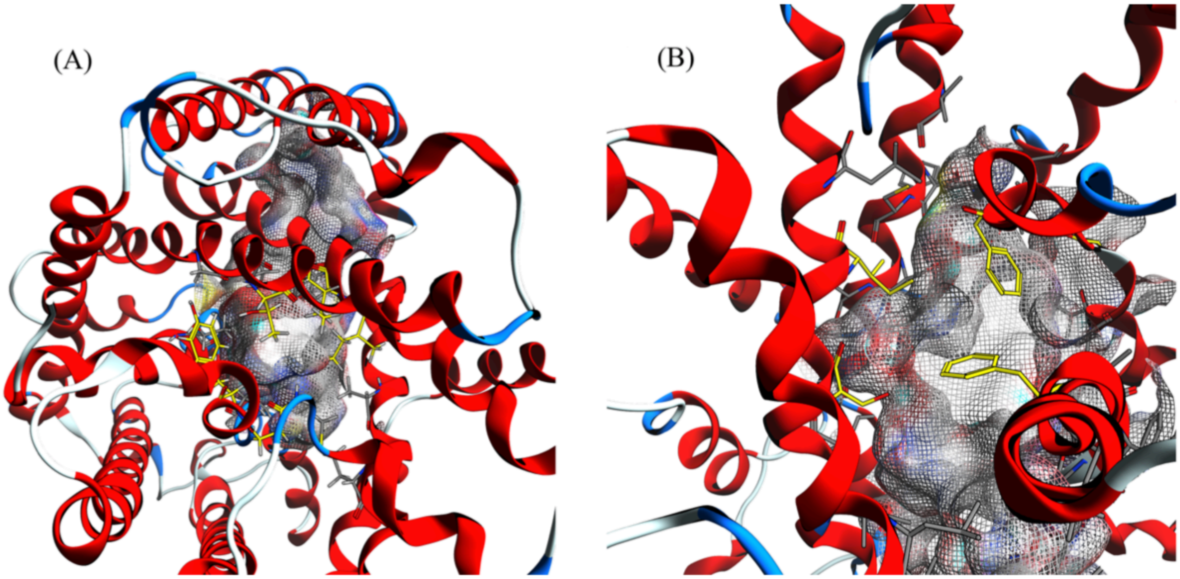

Supplementary Figure 3: Gating residues of LAT1 OF and IF models. (A) residues involved in outward- facing channel closure (Tyr259, Thr345 and Asn258) in OF model; (B) residues involved in inward- facing channel closure (Ile147, Phe252, Ser143 and Phe69) in IF model.

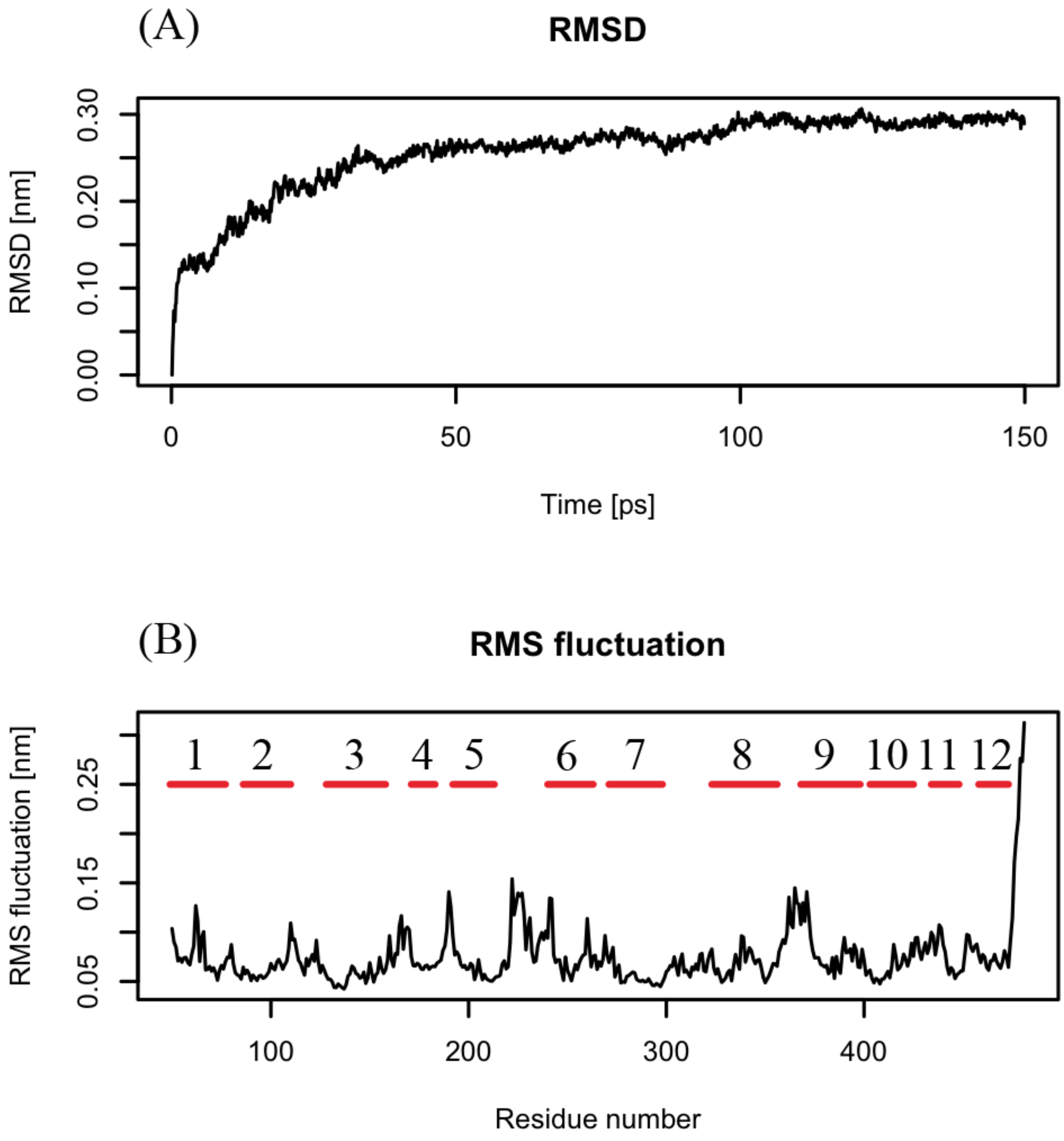

Supplementary Figure 4: Evolution of LAT1 during MD simulation. Panel (A) shows RMSD value over time for LAT1; panel (B) shows the RMS fluctuation, expressed in nm, computed for the α-carbons of LAT1. α-Helices are represented as red lines. C-terminus has the highest mobility, whereas α-helices are stiffer.

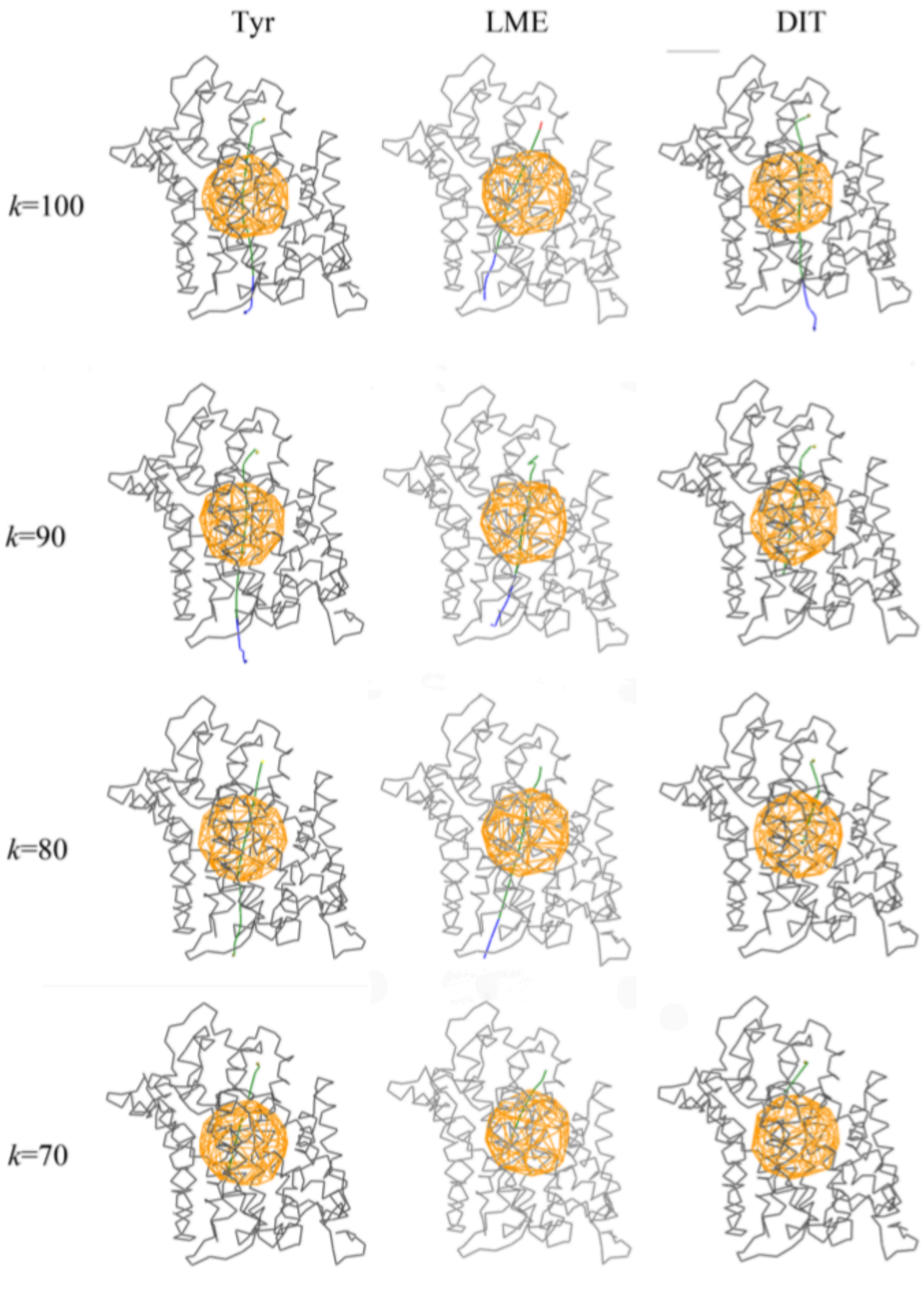

Supplementary Figure 5: tMD paths of LAT1 solutes and inhibitor along the transport channel. Green lines represent solute paths traced *via* Aqua-Duct through a spherical zone of radius 6 Å, centered on selected residues (orange sphere).
